# The Mutual Influences between Depressed *Macaca fascicularis* Mothers and Their Infants

**DOI:** 10.1371/journal.pone.0089931

**Published:** 2014-03-05

**Authors:** Qinming Zhou, Fan Xu, Qingyuan Wu, Wei Gong, Liang Xie, Tao Wang, Liang Fang, Deyu Yang, Narayan D. Melgiri, Peng Xie

**Affiliations:** 1 Institute of Neuroscience, Chongqing Medical University, Chongqing, China; 2 Chongqing Key Laboratory of Neurobiology, Chongqing, China; 3 Department of Neurology, The First Affiliated Hospital of Chongqing Medical University, Chongqing, China; 4 Yongchuan Hospital of Chongqing Medical University, Chongqing, China; Xi'an Jiaotong University School of Medicine, China

## Abstract

**Objective:**

To assess the influence of infant rearing on the behavior of depressed adult female *Macaca fascicularis* and the influence of depressed infant-rearing adult female *Macaca fascicularis* on their infants in a free enclosure environment.

**Methods:**

Here, 20 depressed subjects and then 20 healthy subjects were randomly selected from a total population of 1007 adult female *Macaca fascicularis* subjects. Four depressed subjects and eight healthy subjects were rearing infants. By focal observation, three trained observers video-recorded the selected subjects over a total observational period of 560 hours. The video footage was analyzed by qualified blinded analysts that coded the raw footage into quantitative behavioral data (i.e., durations of 53 pre-defined behavioral items across 12 behavioral categories) for statistical analysis.

**Results:**

Between infant-rearing and non-rearing healthy subjects, ten differential behaviors distributed across five behavioral categories were identified. Between infant-rearing and non-rearing depressed subjects, nine behaviors distributed across five behavioral categories were identified. Between infant-rearing healthy and infant-rearing depressed subjects, fifteen behaviors distributed across six behavioral categories were identified.

**Conclusion:**

Infant-rearing depressed adult female *Macaca fascicularis* subjects may have a worse psychological status as compared to non-rearing depressed counterparts. Infant rearing may negatively influence depressed *Macaca fascicularis* mothers. Infant-rearing depressed subjects were less adequate at raising infants as compared to infant-rearing healthy subjects. Thus, maternal depression in this macaque species may negatively impact infatile development, which is consistent with previous findings in humans.

## Introduction

Depression is a common psychiatric disorder across children, adolescents, adults, and the elderly. Women are particularly at risk for developing depression due to a complex set of social, psychological, and biological reasons. A full 10%–20% of women present with depressive symptoms [1], [2]. Moreover it has been reported that approximately 20%–25% of women display heightened depressive symptoms during both pregnancy and the post-partum period [3]. Now postpartum depression has become a focus of concern. Postpartum depression may lead to neglect of the child, family breakdown, self harm, and suicide [4]. Although the frequency, severity, and family consequences of postpartum depression have been extensively investigated [5]–[7], the question of whether there are differences between postpartum depression and non-postpartum depression is still unclear. In the previous studies, the agreement on the issue can't be arrived. Some studies held that the risk of depression after childbirth was higher than that at other times [8]–[10]. Some investigators reported that women after childbirth experienced higher levels of depressive symptomatology than did nonchildbearing women [10], [11]. However, some study reported that there was little to distinguish postpartum depression from non-postpartum depression beyond prevalence and symptom severity [4], [12]–[15]. Even so, the previous research demonstrated that problems related to maternal depression can be transmitted to infants [16]–[20]. Maternal depression has been consistently associated with adverse effects on maternal–infant interactions and some measures of infant development [21].

Macaca fascicularis, also known as the cynomolgus monkey or crab-eating macaca, is a primate species indigenous to tropical southern and SE Asia that taxonomically falls in the parvorder Catarrhini that includes Old World monkeys and higher apes (including humans). M. fascicularis shares strong genetic, neuroanatomical and behavioral homology to man; thus, the species serves as a more suitable experimental model for neuropsychiatric research with a comparably stronger applicability to clinical neuropsychiatry as compared to non-primate behavioral models (e.g., rodents, canines). As a result, behavioral scientists have displayed increasing interest in M. fascicularis and other Macaca species in recent years [22]–[29]. However, there have been no studies on M. fascicularis as a model of maternal depression, so it is not clear whether there are differences between postpartum depression and general depression in M. fascicularis. Although it has been proved that maternal style can affect the physical and cognitive development of infants in several primate species [30]–[32], it is still unclear whether maternal depression affects infantile development in M. fascicularis.

Here, we compared the behaviors of infant-rearing and non-rearing depressed adult female *M. fascicularis* subjects. Then, we compared the infant rearing behavioral patterns between healthy and depressed mothers. We found that infant rearing may exacerbate maternal depression. *M. fascicularis* after childbirth may have higher level of depressive symptomatology than do non-rearing *M. fascicularis*. And according to our study, consistent with humans, maternal depression may impact the quality of parenting and infantile development.

## Methods

### Study Site

This study was conducted from November 2010 to May 2012. The *M. fascicularis* feeding Base of Zhongke Experimental Animal Co., Ltd. (hereinafter “the Company”) is located in Suzhou, P.R.C. at E 31°07′03″ to 31°07′06″, N 120°19′08″ to 120°19′15″. The site is located in a temperate subtropical monsoon maritime climate with an annual average temperature of 19.7°C. The Company imported the *M. fascicularis* subjects from Guangdong and Vietnam in 1990 and established a domestication and breeding base for these monkeys. More detailed information on the study site is provided in the addendum File S1 in Supporting Information.

### Subject Housing and Selection

All *M. fascicularis* subjects were supplied by an operating subsidiary of the Company, the Institute of Laboratory Animal Co., Ltd. (license number SYXK (Su) 2002-0032). All subjects were housed in a free enclosure environment measuring 12 m×4 m×3 m with the wild male-to-female ratio of 1∶8–10. Fisher's exact test showed no statistical differences in this ratio across the free enclosures (*P*>0.01). All free enclosures were consistently maintained on a 12 h light/dark cycle. All subjects were given water *ad libitum*, and fed twice daily with fresh fruit, vegetables and compound high-nutrition monkey food. Meal amounts were adjusted daily according to group needs. Daily cleanings were performed every morning at 7:30 a.m. to prevent biowaste accumulation. If an individual subject displayed abnormal behavior, the veterinarian on call was consulted immediately. More detailed information on the environmental conditions can be accessed in the addendum File S1 in the Supporting Information.

Shively *et al.* first presented the concept that *M. fascicularis* subjects that have been observed with depressed posture may be classified as behaviorally depressed [33]. This behavior was defined as a slumped or collapsed body posture with the head at or below shoulders, in which the animal's eyes are open, yet the animal lacks interest or responsiveness to environmental events [34], [35]. According to this construct, in two weeks observation we selected 20 behaviorally depressed female *M. fascicularis* subjects who were ever observed in the depressed posture from a total population of 1007 subjects. Then, we randomly selected 20 female healthy controls from the same population, all which were shown to be disease-free by veterinary examination. These selected subjects were distributed across different free enclosures. In the selected female behaviorally depressed monkeys, four depressed subjects were rearing infants (infant-rearing depressed group), while the remaining sixteen depressed subjects were not rearing infants (non-rearing depressed group). In the twenty healthy controls, eight healthy subjects were rearing infants (infant-rearing healthy group), while the remaining twelve healthy subjects were not rearing infants (non-rearing healthy group). All subjects displayed normal menstruation. Their age ranged from 9 to 16 years old. The t test showed no statistical differences between the age of the healthy subjects and that of the depressed subjects. According to the archival files provided by Zhongke Experimental Animal Co., Ltd., thirty-five of forty selected subjects had more than one infant rearing experience. Especially, all the mothers in our study reared more than two infants before.

### Observation

From October 2010 and August 2011, recorded observations were divided into seven (7) time phases with 30 minutes allotted per phase. This totals 3.5 hours per day by a total of 120 days, equaling a total recorded observation period of 560 hours. Observations started at 10:00 A.M. with three morning time phases (A2, A3, and A4), and resumed at 2:30 P.M. with four afternoon time phases (P2, P3, P4, and P5). Focal observation of the subjects was conducted by three trained observers, each with a high-definition digital camcorder (SONY DCR-SR43, 1100M).

All video footage was then converted into a viewable format, and analyzed by the behavioral analysis software The Observer XT 10, NOLDUS. Three blinded analysts then randomly analyzed the video footage and coded the behaviors into NOLDUS. The Fleiss' kappa showed an inter-observer reliability exceeding 85% (κ>0.85) for each behavioral item.

### Behavioral Definitions and Categorization

We have constructed and validate a systematic ethogram of the species in a free enclosure [36]. Fifty-three discrete behavioral items grouped across 12 behavioral categories were recorded in NOLDUS; these 12 behavioral categories are briefly defined below. For the included behaviors of every behavior category, see table 1. For precise definitions of all 53 behavioral items, see the addendum File S3 in the Supporting Information section.

**Table 1:** The Included Behaviors of 12 Behavior Categories.

Ingestion behavior	Thermo-regulatory behavior	Rutting and estrous behavior
Feeding while hanging	Huddling	Licking genital area
Feeding while sitting	Quivering	Presenting buttocks
Drinking	Embracing	
Chewing		
Licking residue from floor		
Eating object from body		
Picking remaining food		
Feeding while perching		

Mating behavior	Resting behavior	Parental behavior
Mounting	Sitting on floor	Nursing infant
Copulating	Sitting on floor facing wall	Holding infant
	Perching on shelf	
	Lying on floor	
	Hanging on window or door	
	Hanging on skylight	
	Hanging on iron chain	
	Hanging on ventilator	
	Sitting and sleeping	

Amicable behavior	Conflict behavior	Vigilance behavior
Grooming	Driving	Shifting position
Being groomed	Attacking	Alarmed jumping
	Fleeing	Watching company
	Biting	Miscellaneous calling
	Threatening	
	Being threatened	
	Being attacked	

Communication behavior	Locomotion behavior	Miscellaneous behavior
Lip smacking	Walking on shelf	Shaking body
	Quadrupedal walking on floor	Playing
	Climbing	Licking hair
	Walking on iron chain	Scratching by hind leg
	Walking on skylight	Scratching by foreleg
	Standing	Yawning
		Digging anus
		Rubbing palm on floor
		Licking tail
		Shaking ID card

Ingestion behavior: taking food material or fluids into the subject's digestive system.

Thermo-regulatory behavior: regulating body heat through movement, such as huddling, quivering, embracing or being embraced by another subject.

Rutting and estrous behavior: arousing sexual interest in another subject, such as presenting buttocks.

Mating behavior: sexual intercourse between opposite-sex subjects, such as mounting, copulating.

Resting behavior: refraining from activity.

Parental behavior: providing supervision and care to offspring.

Amicable behavior: providing friendly actions towards another subject, such as grooming.

Conflict behavior: struggling between subjects resulting from incompatible or opposing demands.

Vigilance behavior: responding to threatening external stimuli.

Locomotive behavior: engaging in motile activity (as opposed to resting behavior).

Communication behavior: transmitting information to other subjects via gestures or emitting sounds

Miscellaneous behavior: a set of defined miscellaneous actions, such as scratching.

### Statistical Analysis

The duration (in seconds) of all observable behavioral items were recorded. The average daily durations per subject on both an itemized and categorical basis were calculated as mean ± standard error. Prior to further statistical analysis, the Shapiro-Wilk test was employed to assess asymmetrical distribution in non-zero data sets. For non-normally distributed data sets, the non-parametric Wilcoxon–Mann–Whitney two-sample rank-sum test with a significance level of α = 0.05 was used to detect significant differences in the behavioral items between the experimental groups. For normally distributed behavioral items, the Student's *t*-test was applied to detect significant differences between the experimental groups. All data processing, data management, and statistical analysis were performed using SAS 9.13 software.

### Ethics Statement

The research complied with protocols approved by the Committee on the Ethics of Animal Experiments at Chongqing Medical University, adhered to the legal requirements of the People's Republic of China, and adhered to the American Society of Primatologists (ASP) Principles for the Ethical Treatment of Non-Human Primates. Animal care and housing procedures were in compliance with Chinese regulatory requirements (see addendum File S1 in supporting information) and AAALAC statements. In addition, this study was performed in strict accordance with the recommendations in the Guide for the Care and Use of Laboratory Animals of the Institute of Neuroscience of Chongqing Medical University (Approval No: 20100031). All procedures described were observational under normal rearing circumstance and did not involve physical manipulation of the subjects or changes to their environment or diet.

In brief, complete animal husbandry and veterinary care was provided daily. Animals were fed a nutritious standardized diet, supplemented daily with fresh fruits and vegetables. Animals had unrestricted access to potable water. Animal enclosures were cleaned daily. Animals were observed daily by trained care-takers. Any observed abnormality, disease, or injury was reported to the veterinary staff for diagnosis and treatment. This veterinary support was documented in both hardcopy and electronic formats.

## Results

### Significant Differences Between Groups

In order to statistically distinguish the differential behaviors between infant-rearing and non-rearing healthy subjects and between infant-rearing and non-rearing depressed subjects, the Mann-Whitney U test was applied to rank sum the average daily durations of all 53 recorded behaviors in the aforementioned 12 behavioral categories. Between infant-rearing and non-rearing healthy subjects, ten differential behaviors distributed across five behavioral categories were significantly identified (Table 2). Between infant-rearing and non-rearing depressed subjects, nine behaviors distributed across five behavioral categories were significantly identified (Table 3). The means and standard errors of the average daily durations per subject for both comparisons are provided (Tables 2, 3). We then compared the differences between infant-rearing healthy and infant-rearing depressed subjects by the same methods; fifteen behaviors distributed across six behavioral categories were identified. The means and standard errors of the average daily durations per subject for this comparison are detailed also provided (Table 4).

**Table 2:** Differential Behaviors Between Non-rearing and Infant-rearing Healthy Subjects.

Category	Behavior	Average Duration†	
		Non-rearing healthy subjects*	Infant-rearing healthy subjects*
Rest	Sitting on floor	956.67±605.19*	973.40±564.17*
	Lying on floor	8.08±44.90*	10.10±51.19*
	sitting on floor facing wall	4.65±33.77*	5.91±38.59*
	Perching on shelf	254.57±464.76*	274.89±446.08*
Amicable	Grooming	69.92±176.96*	86.56±189.57*
Locomotion	Climbing	14.85±25.35*	13.06±20.78*
	quadrupedal walking on floor	61.85±63.68*	78.06±71.91*
	Walking on shelf	4.02±10.29	5.93±13.26
Ingestion	Drinking	4.33±19.01*	6.41±23.92*
Thermo-regulatory	Embracing	347.73±509.16*	252.33±446.21*

†Average duration expressed as mean±SD (seconds per phase).

*P<0.05, Mann-Whitney U testing applied to compare groups.

**Table 3:** Differential Behaviors Between Non-rearing and Infant-rearing Depressed Subjects.

Category	Behavior	Average Duration†	
		Non-rearing depressed subjects*	Infant-rearing depressed subjects*
Rest	Hanging on window or door	160.17±384.36*	175.81±384.73*
	Hanging on skylight	12.92±111.09*	15.71±123.55*
	Perching on shelf	225.80±443.57*	265.14±472.73*
Amicable	Grooming	63.98±174.15*	62.26±160.53*
	Being groomed	75.82±169.89*	62.18±136.84*
Locomotion	Walking on skylight	3.28±17.90*	3.65±19.61*
	quadrupedal walking on floor	61.06±60.45*	67.46±66.88*
Ingestion	Drinking	3.47±17.37*	4.80±19.17*
Thermo-regulatory	Embracing	382.96±519.04*	328.23±491.73*

†Average duration expressed as mean±SD (seconds per phase).

*P<0.05, Mann-Whitney U testing applied to compare groups.

**Table 4:** Differential Behaviors Between Infant-rearing Healthy and Infant-rearing Depressed Subjects.

Category	Behavior	Average Duration†	
		Infant-rearing healthy subjects*	Infant-rearing depressed subjects*
Parental	Holding infant	228.00±454.50*	272.20±505.39*
	Nursing infant	18.58±70.30*	17.61±68.44*
Rest	Sitting on floor	973.40±564.17*	964.00±601.95*
	Perching on shelf	274.89±446.08*	265.14±472.74*
	Lying on floor	10.10±51.19*	5.36±30.19*
	Hanging on iron chain	30.78±142.65*	14.31±104.30*
	Hanging on window or door	105.82±292.95*	175.81±384.73*
	Hanging on skylight	1.20±13.12*	15.71±123.55*
Locomotion	quadrupedal walking on floor	78.06±71.91*	67.46±66.88*
	Climbing	13.06±20.78*	14.81±25.37*
	Walking on skylight	1.36±5.78*	3.65±19.61*
Amicable	Grooming	86.56±189.57*	62.26±160.53*
Miscellaneous	scratching by hind leg	2.23±4.87*	2.97±5.64*
	digging anus	1.84±11.72*	1.38±8.57*
Thermo-regulatory	Embracing	252.33±446.21*	328.23±491.73*

†Average duration expressed as mean±SD (seconds per phase).

*P<0.05, Mann-Whitney U testing applied to compare groups.

### Resting Behavior

Between non-rearing and infant-rearing healthy subjects, there were significant differences in four resting behaviors: ‘sitting on floor,’ ‘lying on floor,’ ‘sitting on floor facing wall,’ and ‘perching on shelf’ (Table 2). Between non-rearing and infant-rearing depressed subjects, there were significant differences in three resting behaviors: ‘hanging on window or door,’ ‘hanging on skylight,’ and ‘perching on shelf’ (Table 3). Between infant-rearing healthy and infant-rearing depressed subjects, there were significant differences in six resting behaviors: ‘sitting on floor,’ ‘perching on shelf,’ ‘lying on floor,’ ‘hanging on iron chain,’ ‘hanging on window or door,’ and ‘hanging on skylight’ (Table 4).

### Locomotion Behavior

Between non-rearing and infant-rearing healthy subjects, there were significant differences in three locomotion behaviors: ‘climbing,’ ‘walking on shelf’ and ‘quadrupedal walking on floor’ (Table 2). Between non-rearing and infant-rearing depressed subjects, there were significant differences in two behaviors: ‘walking on skylight’ and ‘quadrupedal walking on floor’ (Table 3). Between infant-rearing healthy and infant-rearing depressed subjects, there were significant differences in three behaviors: ‘quadrupedal walking on the floor,’ ‘climbing,’ and ‘walking on skylight’ (Table 4).

### Amicable Behavior

One amicable behavior, ‘grooming for others,’ was found to be of significantly greater duration in infant-rearing healthy subjects compared to non-rearing healthy subjects (Table 2). Between non-rearing and infant-rearing depressed subjects, two behaviors – ‘grooming’ and ‘being groomed’ – were found to be of significantly lesser duration in infant-rearing depressed subjects (Table 3). Between infant-rearing healthy and infant-rearing depressed subjects, the ‘grooming for others’ behavior was found to be of significantly greater duration in infant-rearing healthy subjects (Table 4).

### Ingestion Behavior

One ingestion behavior, ‘drinking,’ was found to be of significantly greater duration in both infant-rearing groups relative to their non-rearing counterparts (Tables 2, 3). Between infant-rearing healthy and infant-rearing depressed subjects, there were no significant differences in ingestion behavior (Table 4).

### Thermo-regulatory Behavior

One thermo-regulatory behavior, ‘embracing,’ was significantly differentiated in all three comparisons. The embracing behavior was found to be of significantly lesser duration in both infant-rearing groups relative to their non-rearing counterparts (Tables 2, 3). Moreover, the ‘embracing’ behavior in infant-rearing healthy subjects had a significantly lesser duration than infant-rearing depressed subjects (Table 4).

### Parental Behavior

Two parental behaviors, ‘holding infant’ and ‘nursing infant,’ were statistically differentiable between infant-rearing healthy subjects and infant-rearing depressed subjects (Table 4). ‘Holding infant’ was found to be of significantly greater duration in infant-rearing depressed subjects relative to infant-rearing healthy subjects, while ‘nursing infant’ was found to be of significantly greater duration in infant-rearing healthy subjects relative to infant-rearing depressed subjects (Table 4).

### Miscellaneous Behavior

There were significant differences in two miscellaneous behaviors, ‘scratching by hind legs’ and ‘digging anus,’ between infant-rearing healthy subjects relative to infant-rearing depressed subjects. ‘Scratching by hind legs’ as found to be of significantly greater duration in infant-rearing depressed subjects relative to infant-rearing healthy subjects, while ‘digging anus’ was found to be of significantly greater duration in infant-rearing healthy subjects relative to infant-rearing depressed subjects (Table 4).

## Discussion

After applying the Mann-Whitney U test to determine significant differences between infant-rearing and non-rearing healthy subjects, ten behaviors across five behavioral categories were revealed to be significantly distinguishable. Likewise, between infant-rearing and non-rearing depressed subjects, nine behaviors of five behavioral categories were found to be significantly differentiable. In addition, there were fifteen significantly differentiated behaviors across six categories between infant-rearing healthy subjects and infant-rearing depressed subjects.

### The Influence of Infant Rearing on Depressed Mothers

In human there is argue whether there are any differences between postpartum depression and general depression at other time. In the present study we compared the behaviors of infant-rearing and non-rearing depressed adult female *M. fascicularis* subjects to explore the differences of depression in two groups.

With respect to resting and locomotive behaviors, infant-rearing healthy subjects were observed to rest and move in more comfortable areas (‘sitting on floor’, ‘lying on floor’, ‘sitting on floor facing the wall’, ‘perching on shelf’, ‘walking on shelf’, and ‘quadrupedal walking on floor’) as compared to non-rearing healthy subjects (‘climbing on window or door’). Based on our daily field observations of this captive macaque species in a free enclosure environment [36], higher status monkeys (give more aggression, receive more submission) tend to rest in more comfortable positions (e.g., floor, shelf, iron chain) as opposed to lower status monkeys (receive more aggression, present more submission), which tend to rest in more uncomfortable places (e.g., window, door, skylight). This finding suggests that infant rearing in healthy subjects may improve social status. Previous work has also shown that a female macaque's social status is positively influenced by possessing an infant [37], [38]. Meanwhile, infant-rearing depressed subjects were more frequently observed resting and moving in less comfortable areas (‘hanging on window or door,’ ‘hanging on skylight,’ and ‘walking on skylight’) than non-rearing depressed subjects. Thus infant-rearing depressed mothers may have lower status than non-rearing depressed mothers. Willard *et al.* and Michopoulos *et al.* have previously demonstrated that socially subordinate adult female *M. fascicularis* subjects more commonly present depression-like behavior and dysregulation of the limbic–HPA axis (e.g., decreased cortisol in response to ACTH and decreased glucocorticoid negative feedback; lower CSF DOPAC and serum PACAP) [39], [40]. This finding suggests that infant-rearing depressed subjects may experience decreased social status and elevated stress levels relative to non-rearing depressed subjects, which may increase the risk of depressive behavior. But we can't give a proper explanation that the time the infant-rearing depressed subject spent on ‘perching on shelf’ and ‘quadrupedal walking on floor’ was also significantly longer than non-rearing depressed subject.

With respect to amicable behavior, ‘grooming’ and ‘being groomed,’ were found to be of significantly lower average duration in infant-rearing depressed subjects as opposed to non-rearing depressed subjects. Yet one amicable behavior, ‘grooming,’ was found to be of significantly higher average duration in infant-rearing healthy subjects than non-rearing healthy subjects. ‘Grooming’ and ‘being groomed’ have been extensively investigated as socially affiliative behaviors in macaque species. Grooming behaviors ameliorate social relationships and have been previously associated with higher social status and lower stress levels in macaques. It can not only remove dirt and parasite from the body but also play an important role in the establishment and maintenance of social bonds [41]. It may bring anxiety reduction in both recipient of grooming and donor [42]–[45]. Both ‘grooming’ and ‘being groomed’ were decreased in infant-rearing depressed subjects relative to non-rearing depressed subjects, suggesting a more depressed state on account of infant rearing.

With respect to ingestion and thermo-regulatory behaviors, the significant differential behaviors between infant-rearing and non-rearing depressed subjects were identical to those between infant-rearing and non-rearing healthy subjects. One ingestion behavior, ‘drinking,’ was found to be of significantly higher average duration in the infant-rearing groups as compared to their non-rearing counterparts. As infant-rearing females nurse their infants and engage in more physically demanding behaviors (e.g., preening, walking, and standing), it can be surmised that increased water consumption is a homeostatic compensatory mechanism for fluid loss through lactation and sweating. Accordingly, Morse *et al.* have reported that milk supply increases with fluid intake [46]. One thermo-regulatory behavior, ‘embracing,’ was found to be of significantly lower average duration in the infant-rearing groups relative to their non-rearing counterparts. Infant-rearing subjects require more time to hold and nurse their infants, leaving less time to embrace other subjects.

### The Negative Effects of Depressed Mothers on Infants

Many previous studies have demonstrated that maternal style affects the infants' physical and cognitive development in several primate species [30]–[32]. In our study we compared the healthy mothers' and the depressed mothers' maternal style by their own behavior, and surmised the possible effects on the infants' development.

With respect to parental behavior, the average duration of ‘holding infant’ was significantly higher in infant-rearing depressed subjects relative to infant-rearing healthy subjects. On the contrary, the average duration of ‘nursing infant’ was significantly lower in infant-rearing depressed subjects relative to infant-rearing healthy subjects. With respect to ‘holding infant’ behavior, subjects were observed releasing infants during feeding times. However, if the subject lost eye contact with the infant and/or if the infant left the subject's immediate proximity, the subject would follow in pursuit. The ‘holding infant’ behavior serves to nurture the mother-infant bond while protecting the infant from potential aggressors or injury. The previous studies have introduced a common terminology, protective mothers and rejecting mothers [47], [48]. Protective mothers tend to restrain their infants and maintain contact longer whereas rejecting mothers tend to interrupt the contact with their infants and spend more time at a distance. In our study the depressed mothers were more likely to be protective mothers, which held their infants for a significantly longer duration than infant-rearing healthy subjects. It has been shown that infants who were protected more show less interest in the external environment and more unresponsive to the novel situation [30]. Schino found that infants who were protected more were more fearful and cope worse with stressful situation when adult, and didn't have a good development of independence and a less anxiety personality [49]. Additionally, Maestripieri *et al.* demonstrated infants who were protected more by their mothers had higher cortisol levels than those who were protected less, while infants who were rejected more had lower CSF 5-HIAA than infants who were rejected less [50].

In reference to ‘nursing infant’ behavior, subjects were observed preening infants from head to tail, with a disproportionate focus on inspecting and cleaning the anus. ‘Nursing infant’ behavior appears to keep the infant free from parasitic organisms and/or their respective vectors that preferentially attach to the skin and peri-anal regions. Several parasitic worms (e.g., *Strongyloides*, *Enterobius*) have been noted to infect tropical primates via the epidermal and anal routes, so preening behavior may have evolved to defend against the spread of such infections in primate social groups [51]. In addition to removing foreign debris and organisms, Dunbar *et al.* held that preening and other grooming behaviors provide a social function in primate groups by reducing psychological tension and maintaining amicable relationships [52]. In this context, ‘preening infant’ behavior may serve to socialize the infant. Accordingly, an increased frequency of infant preening has been correlated with larger cub populations and stronger social bonds in wild Tibetan macaques (*Macaca tibetana*) [53]. Here, we found that infant-rearing healthy subjects spent more time nursing their infants than their depressed counterparts. Infant-rearing depressed subjects may have less interest in keeping their infants free from parasitic organisms or other pests and/or lack the capability of developing their infants' social competence.

The average duration of four resting behaviors, ‘sitting on floor,’ ‘perching on shelf,’ ‘lying on floor,’ and ‘hanging on iron chain,’ were significantly lower in infant-rearing depressed subjects as compared to infant-rearing healthy subjects. The average duration of two resting behaviors, ‘hanging on window or door’ and ‘hanging on skylight,’ were significantly higher in infant-rearing depressed subjects as compared to infant-rearing healthy subjects. Therefore, infant-rearing depressed subjects selectively rested in uncomfortable places, perhaps on account of their lower social status. Chance demonstrated that infants of subordinate female had less freedom of movement within the group so that they have less varied experience than infants of dominant female [54]. In addition, the window and skylight did not provide sufficient area to allow infants to move about freely, which may have adverse effects on their development. Thus, as opposed to the infant-rearing healthy subjects, the social environment was not beneficial for infant-rearing depressed subjects in raising their infants. The same reasoning can be applied to locomotive behavior, as ‘quadrupedal walking on floor,’ was found to be of significantly lower average duration, and ‘climbing’ and ‘walking on skylight’ was significantly higher, in infant-rearing depressed subjects relative to infant-rearing healthy subjects.

One amicable behavior, ‘grooming,’ was found to be of significantly lower average duration in infant-rearing depressed subjects relative to infant-rearing healthy subjects. Grooming is a social activity which may aid infants with coordination and social skill development. The increased grooming behavior in infant-rearing healthy subjects is consistent with Poirier *et al.*'s work that manifested play and social activity were more common among infant-rearing and infant primates as compared to solitary adults [55]. However, in infant-rearing depressed subjects, the significant decline in this behavior suggests that infant-rearing depressed subjects do not excel at developing their infants' social ability.

One miscellaneous behavior, ‘scratching by hind leg,’ was of significantly higher average duration in infant-rearing depressed subjects relative to infant-rearing healthy subjects, which may correspond with increased maternal anxiety in infant-rearing depressed subjects. Maternal anxiety is typically expressed through physiological changes (e.g., autonomic nervous system activation, release of catecholamines and cortisol) and behavioral changes such as increased vigilance and increased rates of self-directed activities [56], [57]. Maternal anxiety has also been associated with an increased risk of behavioral disturbances in offspring. However, another miscellaneous behavior, ‘digging anus,’ was found to be of significantly lower average duration in infant-rearing depressed subjects relative to infant-rearing healthy subjects. It is possible that infant-rearing depressed subjects had a decreased interest in cleaning themselves.

One thermo-regulatory behavior, ‘embracing,’ was of significantly higher average duration in infant-rearing depressed subjects relative to infant-rearing healthy subjects. Ventro-ventral embracing is an affiliative and bonding behavioral pattern commonly observed between macaque females living in captivity [58]. Previous studies have exhibited that depression adversely affects affiliative communication and social bonding. Thus, the duration of embracing behavior in infant-rearing depressed subjects should be shorter than that in infant-rearing healthy subjects. However, we observed quite the opposite in infant-rearing depressed subjects here, suggesting that infant-rearing depressed subjects may increasingly rely on others for emotional support.

### Conclusion

Firstly, the study demonstrates that infant-rearing depressed adult female *Macaca fascicularis* subjects may have a worse psychological status as compared to non-rearing depressed counterparts. Infant rearing may negatively influence depressed *Macaca fascicularis* mothers by exacerbating maternal depression. Secondly, infant-rearing depressed subjects were less adequate at raising infants as compared to infant-rearing healthy subjects. Thus, maternal depression in this macaque species may negatively impact infantile development, which is consistent with previous findings in humans. However, a limitation of such a study is that we didn't track the infantile behavior and don't know whether the factors associated with depression clearly. Future studies should be of longer duration and investigate the relationship(s) between behavioral outcomes and molecular physiological changes.

## Supporting Information

File S1 **Ethics Statement on Non-human Primate Research.**
(DOC)

File S2 **Behavioral Definitions and Ethogram Validation.**
(DOC)

File S3 **Raw data.**
(XLSX)
