# Supplementary material for: The Mutual Influences between Depressed Macaca fascicularis Mothers and Their Infants
Source: PLoS One. 2014 Mar 5;9(3):e89931. doi: 10.1371/journal.pone.0089931 (PMC3943858; doi:10.1371/journal.pone.0089931)
Supplement: File S1 — Ethics Statement on Non-human Primate Research. (DOC) [file pone.0089931.s001.doc]

**Ethics Statement on Non-human Primate Research**

***Version: 1.0***

**Issued From Primate Behavior Research Group**

**Institution of Neuroscience**

**Chongqing Medical University Press**

**Oct-1-2013**

Preface

This Ethics Statement on Non-human Primate Research aims to detail the work processes and environment of the *Macaca fascicularis* Behavior Research Group. Under the supervision of feeders at the Zhongke Experimental Animal Co., Ltd., the observers studied the daily habitual activities of *M. fascicularis* subjects such as eating, mating, resting, social interaction, and child-care. After approximately three months of passive observation, the observers took over feeding of the *M. fascicularis* subjects to habituate themselves to the subjects. During the study, the observers remained in a stationary position in front of the free enclosure, and by means of video cameras, recorded the behavior of the subjects. For more detailed information, see [PRC government documents]

[DB44-T 348-2006 Macaca animal husbandry management practices](http://www.freebz.net/downpage.asp?id=133401)

[GB14922.1 level of laboratory animals and monitoring of Parasitology](http://www.freebz.net/downpage.asp?id=67767)

[GB14922.2 laboratory animals and micro-level monitoring](http://www.bysy.edu.cn/dwsyzx/zc/gjbz/30363.shtml)

[GB14925-2001 environment and facilities For Laboratory animals](http://wenku.baidu.com/view/83f6cb46b307e87101f696de.html)

[GB laboratory animals feed monkeys](http://www.bzwxw.com/downloadhtml/05/922523.html)

# Ethics Approval Document

## Purpose

Our aim is to assess the influence of infant rearing on the behavior of depressed adult female *Macaca fascicularis* and the influence of depressed infant-rearing adult female *Macaca fascicularis* on their infants in a free enclosure environment.

## Research Plan and Workflow Chart

In congruence with our purpose, 20 behaviorally depressed female *Macaca fascicularis* subjects were selected from a total population of 1007 subjects, which were from the *M. fascicularis* Feeding Base of the Suzhou Xishan Zhongke Experimental Animal Co., Ltd. (“the Company”). Then 20 female healthy *Macaca fascicularis* subjects were randomly selected (using a random seed of 101217) from the same population. These selected subjects were distributed across different free enclosures and each select subject was marked by gentian violet stain for identification purposes. As there was at most two (2) selected subjects per free enclosure, the observer could quickly identify the selected subjects for observation (as displayed in the following photographs). Furthermore, by means of SAS software, the observation schedule was arranged to account for the work schedule of the Company.

**The key factor in our field observations was not to disturb the subject's daily routine, namely through the observer sitting still in front of the free enclosure to record the subject during each observational phase.** In recording observations, three (3) high-pixel videos were recorded using a SONY 1100 megapixel camcorder. A Lenovo PC was used to transfer and convert the videos into a viewable format for analysis through NOLDUS Observer XT software (version 10.0, Noldus Information Technology, Leesburg, PA) [26]. The list of behaviors was encoded to satisfy the operational requirements of NOLDUS. Three (3) qualified observers blindly watched the videos and used NOLDUS to record data on the frequency and duration of each behavior. Inter-observer reliability between the three (3) observers was determined to be greater than 85% for each behavior.


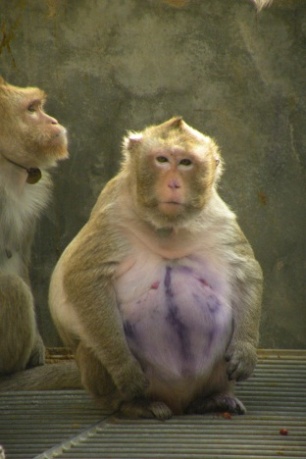

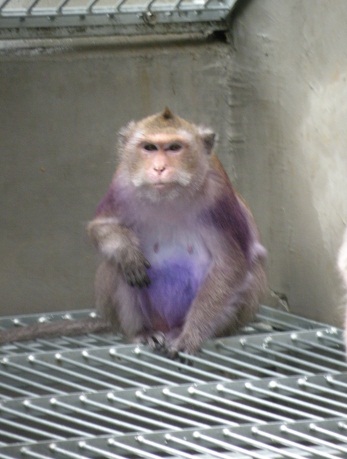

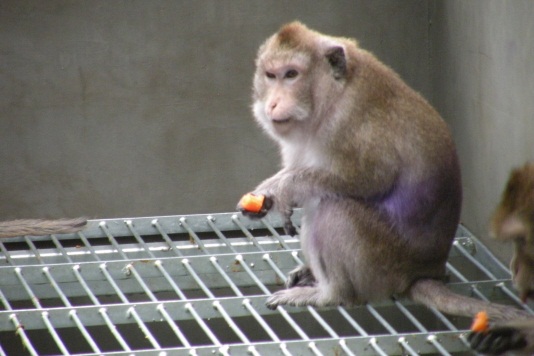

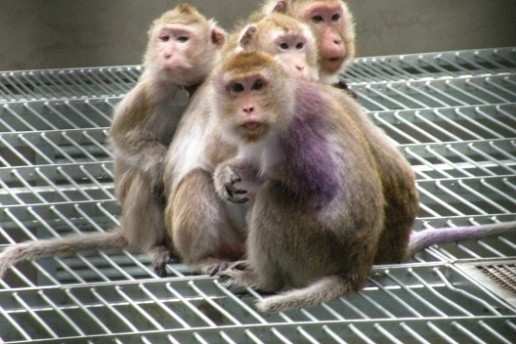

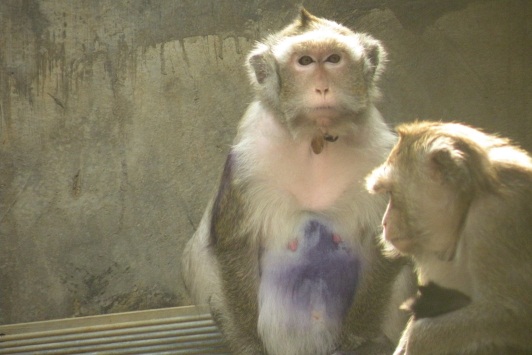


## 1.3 The Ethics Committee of Chongqing Medical University


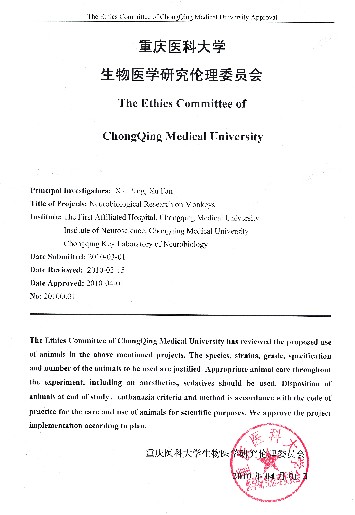
The Ethics Committee of Chongqing Medical University (“the Committee”) reviewed the proposed use of animals in the aforementioned research plan. The species, strains, grade, specification and number of animals to be used were deemed justified by the Committee. Appropriate animal care throughout the experiment (including anesthetic and sedative use when necessary) was recommended. Disposition of animals at the conclusion of the study, euthanasia criteria and method accorded with the code of practice for the care and use of animals for scientific purposes .The Committee approved the project implementation according to the aforementioned research plan. For further details, see Appendix 1.

## 1.4 Emergency Handling Process

In the event of any abnormal phenomenon or emergency situation, the observers would immediately inform the feeder and veterinarian on staff, who would take the appropriate actions.

## 1.5 Observational Conditions

Observational conditions were dictated by the occupational requirements of the company (including staff assignment, breeding management, accommodation, environment, feeding, water and tools), and supporting governmental institutional by-laws (e.g., sanitation and epidemic prevention, health monitoring, and record-keeping).

# 2. Comprehensive Property of the Company

## 2.1 Description of the Company

### 2.1.1 [Introduction of the Company](http://www.szxszk.com/aboutus.do?kind=2)


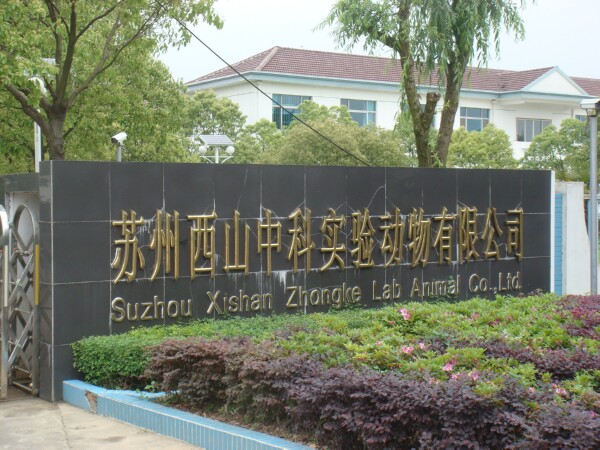
Suzhou Xishan Zhongke Laboratory Animal Co., Ltd. (“the Company”) **(See right )** was founded in 1999 through the exclusive investment of Suzhou Xishan National Modern Agriculture Demonstration Park. The Company covers about 100,000 m^2^ and has approximately 120 employees on staff. It is currently the only non-human primate husbandry and breeding base in East China and houses more than 7000 cynomolgus monkeys, 1000 rhesus monkeys and 1500 Beagle dogs. The Company manages this inventory through animal information and health management systems. Its inventory is widely sold across China, Southeast Asia and North America.


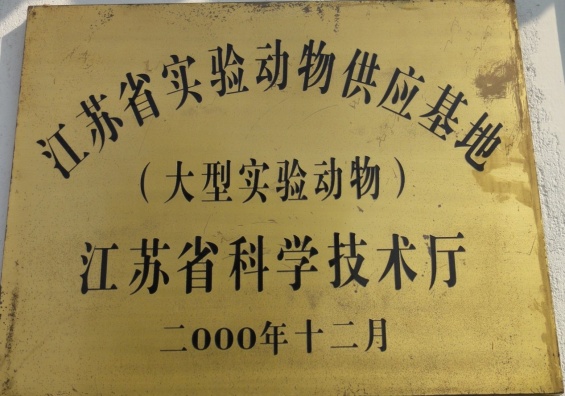
The Company was approved as the ***Large Laboratory Animal Supply Base of Jiangsu Province* (See left)** by the Science and Technology Department of Jiangsu Province in 2001, the ***Animal Experiment Open Laboratory of Jiangsu Province*** in 2002, the ***Animal Experiment Open Service Center of Jiangsu Province*** in 2003. The Company also established the Suzhou Drug Safety Evaluation and Research Center in 2006, which has become a key state GLP laboratory in China. Over the past three years, this center has been actively undertaking several science and technology projects, including the National Innovation Fund, the Natural Science and Technology Fund of Jiangsu Province, Invigorating Trade through Science and Technology project of Jiangsu Province, and several science and technology planning and service industry development projects of Suzhou.

Recently, the Company completed construction of a set of new animal housing facilities, which can house 20,000 additional monkeys. Moreover, a SPF non-human primate colony will be completed shortly and a new internal research center will be finished in 2012.

### [2.1.2 Company Agencies](http://www.szxszk.com/jigou.do)


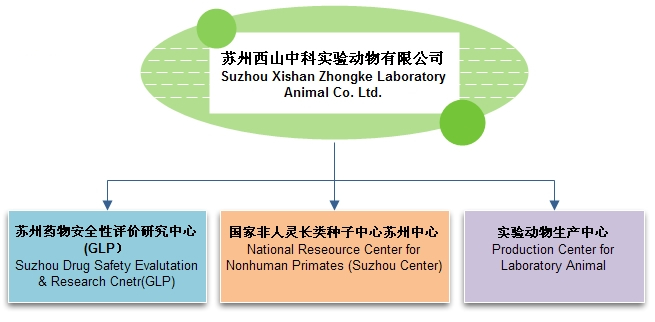


### [2.1.3 Seed Introduction](http://www.szxszk.com/seed.do?id=1)


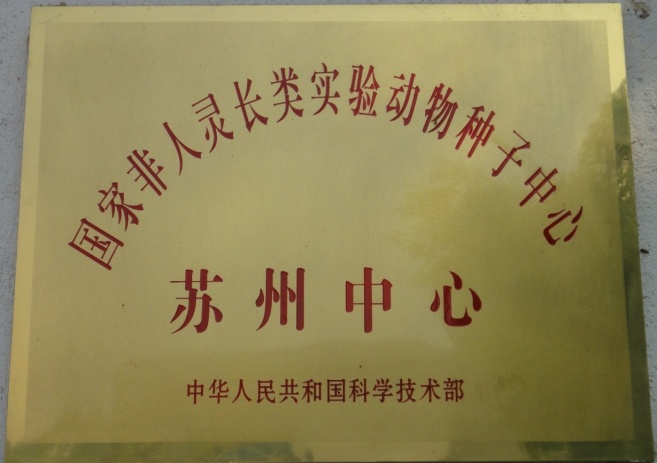
In June 2008,the Company officially issued a document (Zhongkefa (2008) No.6) declaring its intention to establish a ***National Non-human Primate Resource Center*** ("the resource center") in order to i) strengthen studies on conservation and cultivation of non-human primates, ii) identify and breed stable non-human primate species and strains, iii) establish China's first SPF non-human primate population, iv) scientifically protect and manage China’s nonhuman primate resources, and v) provide high-standard nonhuman primates for domestic and international users. In early 2009, the Company applied to the Ministry of Science and Technology for a license **(see below)** and passed the on-site examination and acceptance check conducted by an expert panel from the Ministry of Science and Technology.

The ultimate goals of the resource center are to i) explore, improve and establish non-human primate husbandry, breeding, genetic testing and management standards; ii) set up high-standard ordinary non-human primate colonies and SPF non-human primate closed colonies to better protect China's non-human primate resources; iii) provide comprehensive and integrated high-quality services including non-human primate laboratory resources, husbandry, breeding and conservation technologies and information on the latest research; and iv) provide personnel training for the further development of life sciences, medicine and related disciplines.

## 2.2 Location and Main Facilities <Graphic illustration>


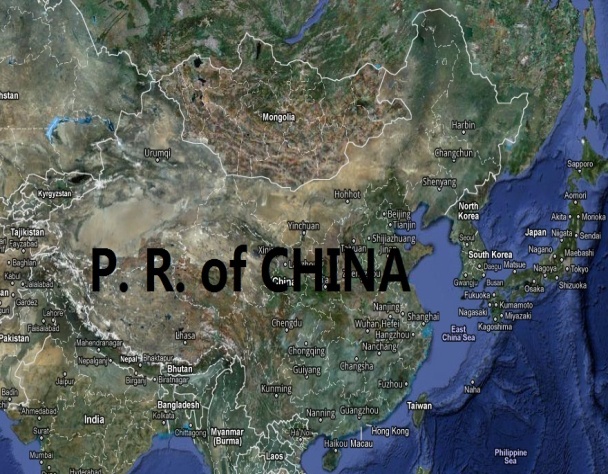

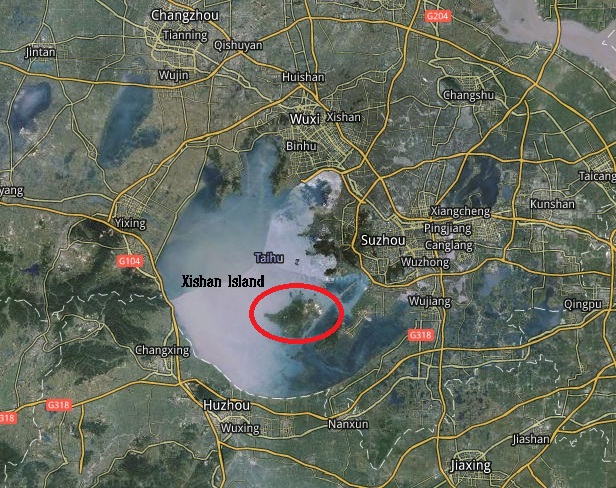


Fig. 1 P.R. of CHINA Fig. 2 Suzhou City-Xishan Island


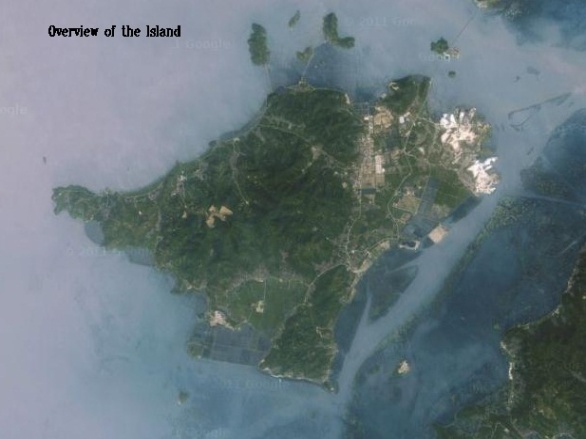

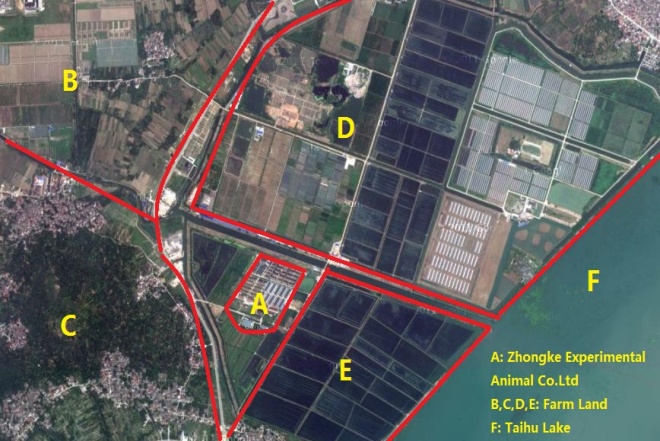


Fig. 3 Satellite view of Xishan Island Fig. 4 Map of the Company & surrounding area


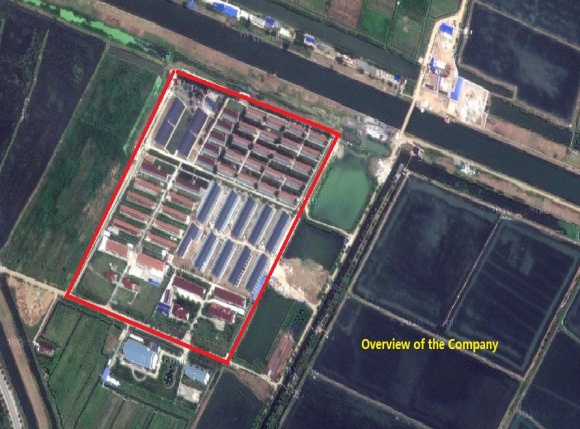

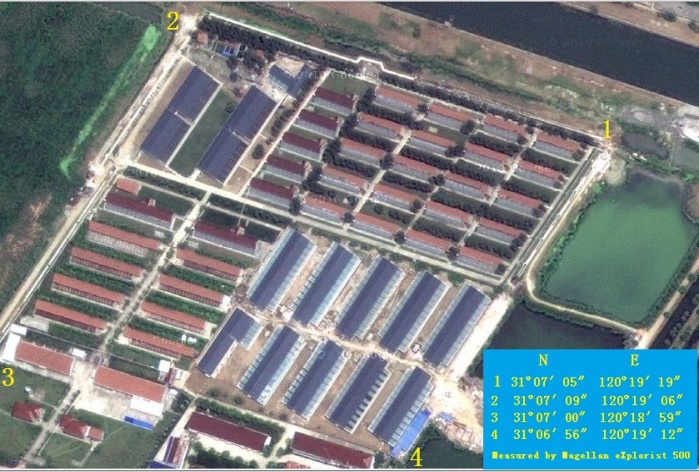


Fig. 5 Satellite view of the Company Fig. 6 View of the Company (with GPS data)


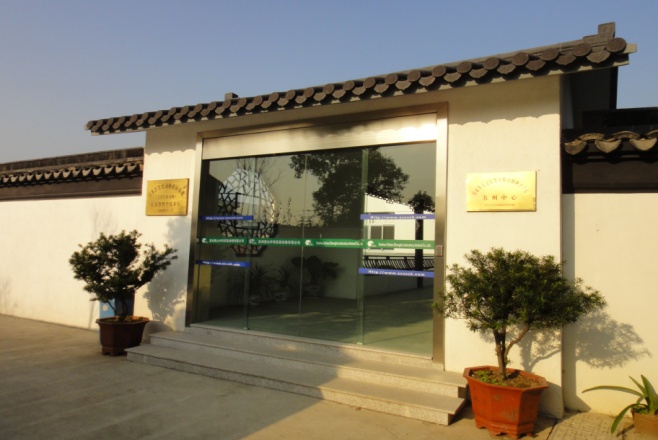

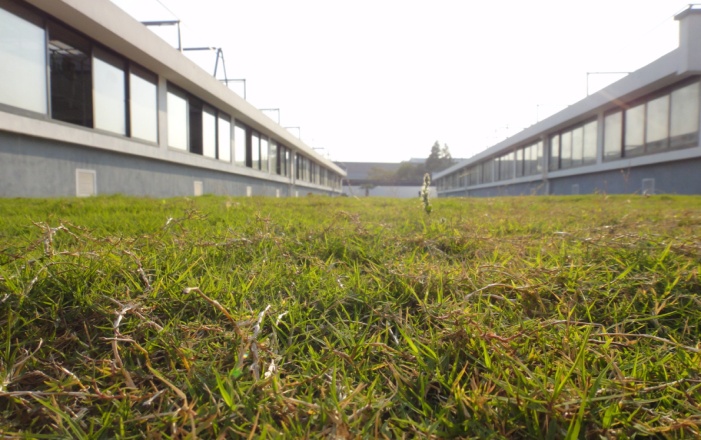


Fig. 7 View of front entrance Fig. 8 Interior greenery


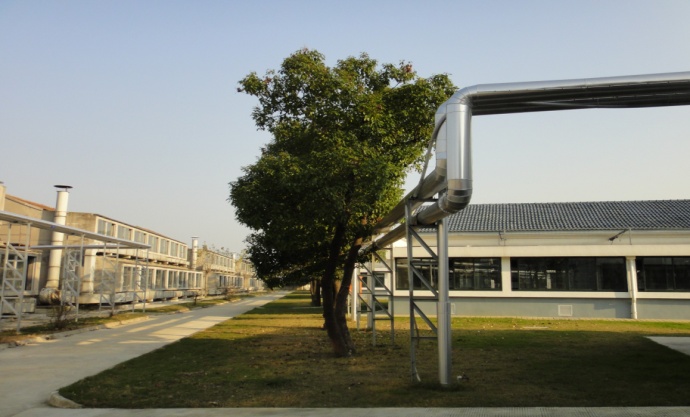

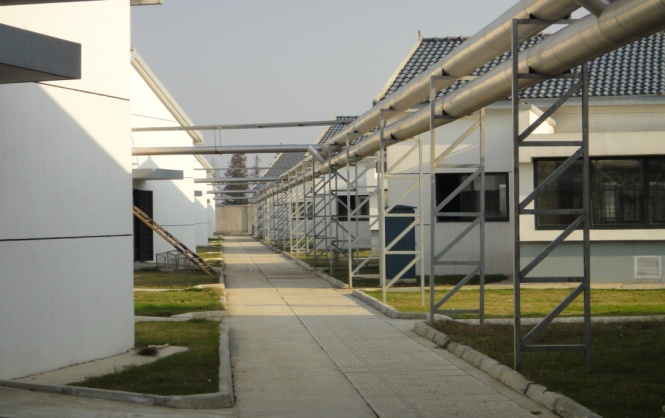


Fig. 9 Interior view Fig. 10 Exterior view of free enclosure facilities


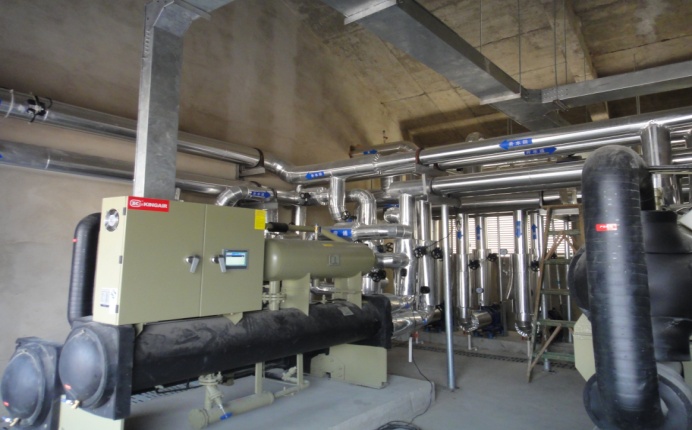

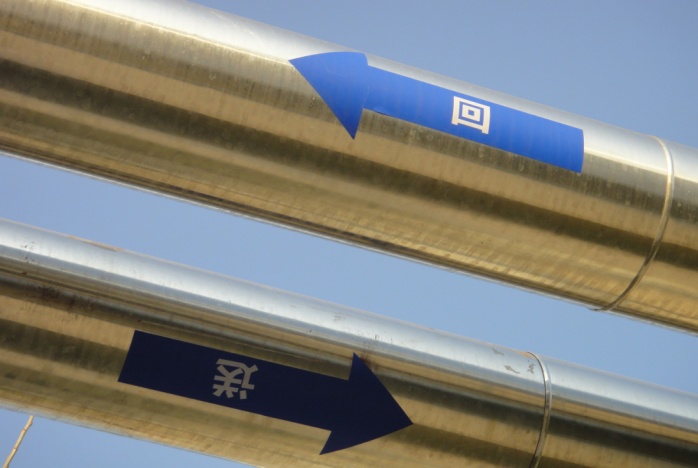


Fig. 11 Central A/C unit Fig. 12 Heating pipes


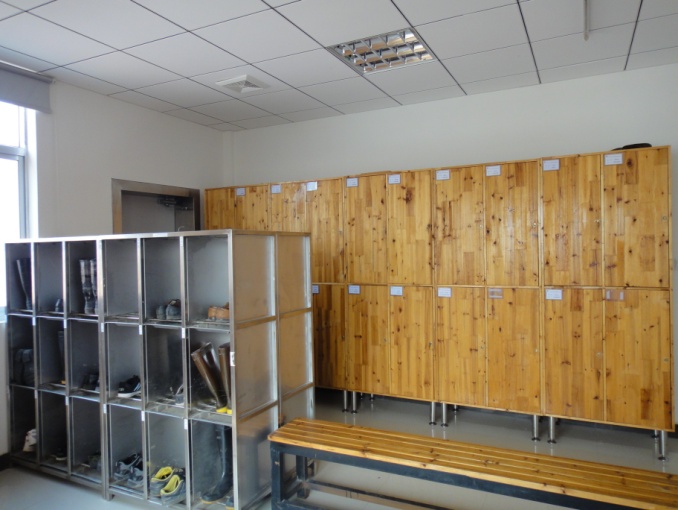

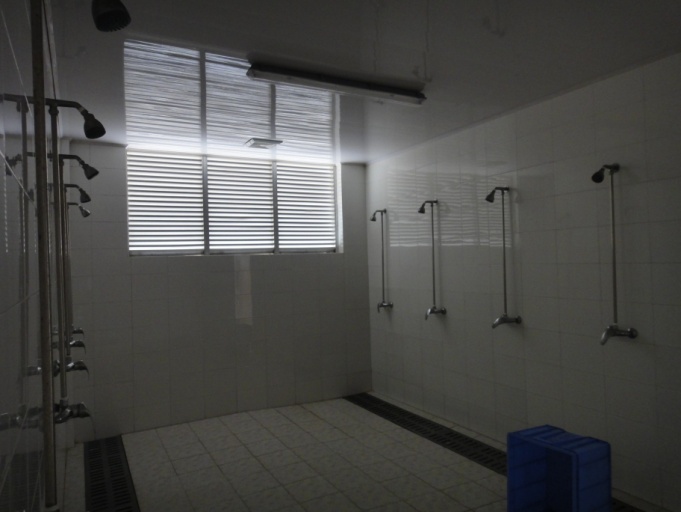


Fig. 13 Changing room Fig. 14 Shower room


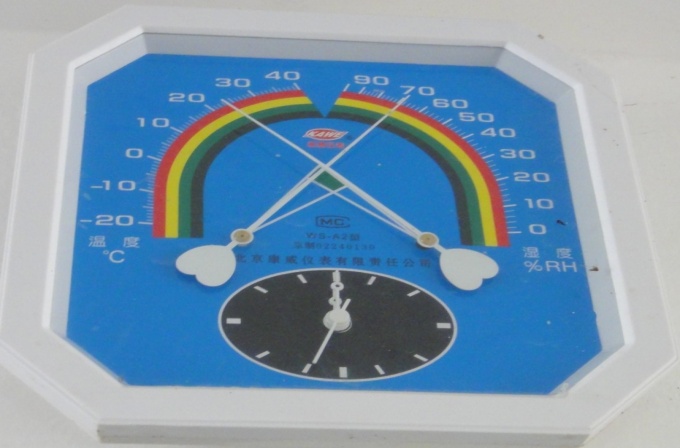

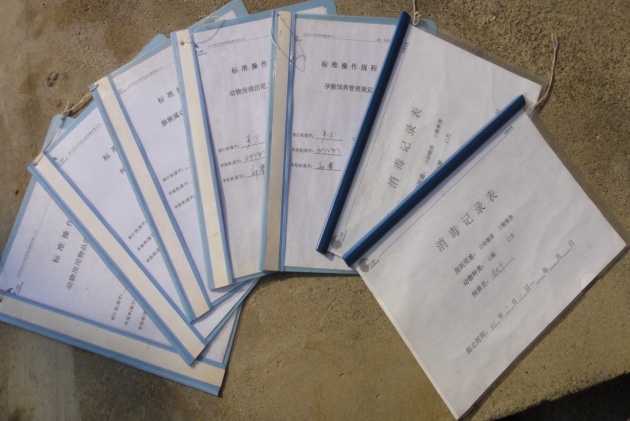


Fig. 15 Thermograph and hygrometer Fig. 16 Staff manuals


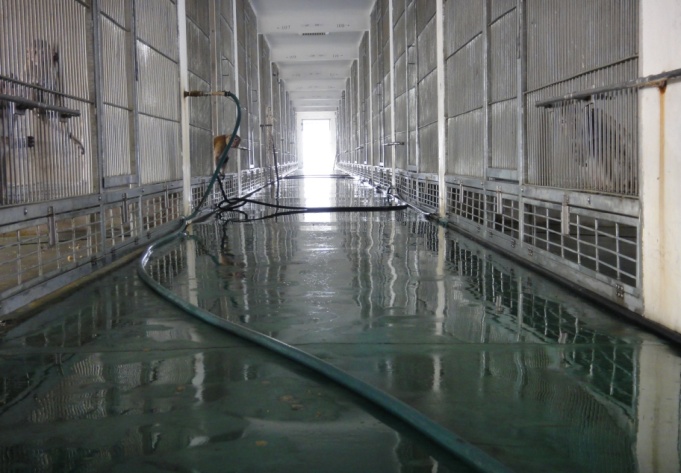

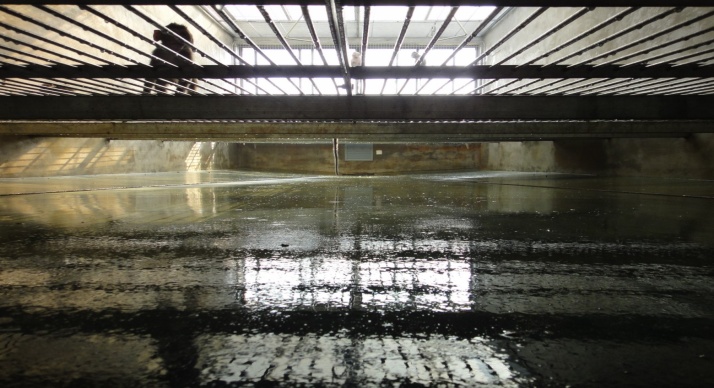


Fig. 17 Interior view of free enclosure facility Fig. 18 Ground view of free enclosure

## Completed Set of Legal Approval Documents

### 2.3.1 National Key Protected Wild Animal Domestication and Breeding License


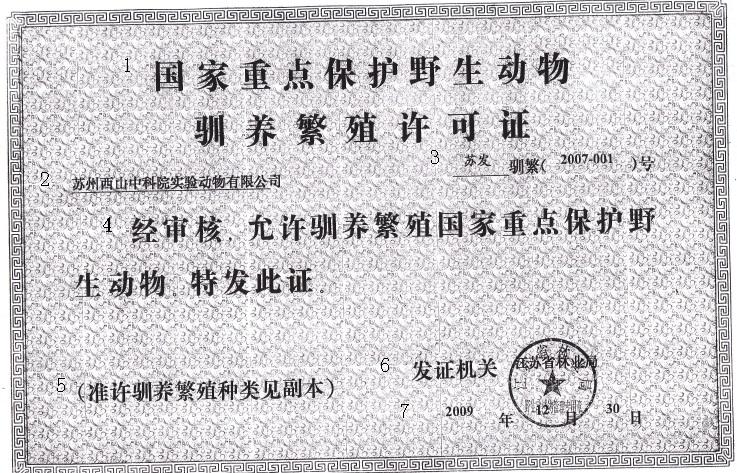


| **Item** | **English translation** |
| --- | --- |
| 1 | National Key Protected Wild Animal Domestication and Breeding License |
| 2  2 | Suzhou Xishan Zhongke Laboratory Animal Co., Ltd. |
| 3 | Suzhou Province Issue: Feed No. 2007-001 |
| 4 | Audited and permitted to domesticate and breed national key protected wild animals |
| 5 | Allowed to distribute copies of this National Key Protected Wild Animal Domestication and Breeding License |
| 6 | Issuing authority: Forestry Bureau of Jiangsu Province |
| 7 | Date: December 30, 2009 |

### 2.3.2 Animal Epidemic Prevention Certificate


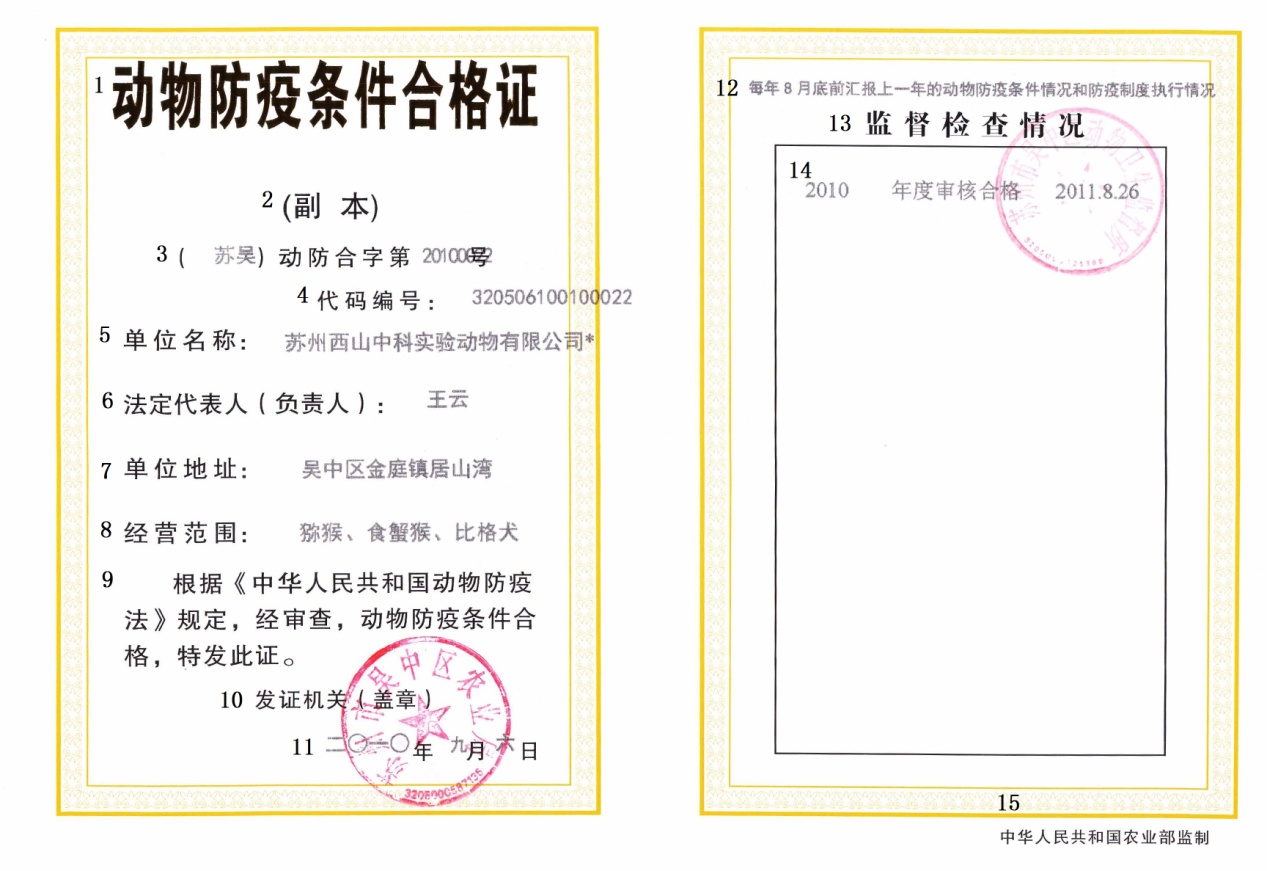


| **Item** | **English translation** |
| --- | --- |
| 1 | Animal Epidemic Prevention Certificate |
| 2 | Copy |
| 3 | Suzhou Province Animal Prevention No.: [see # above] |
| 4 | Code number: [see # above] |
| 5 | Name: Suzhou Xishan Zhongke Laboratory Animal Co., Ltd. |
| 6 | Legal representative: Yun Wang |
| 7 | Address: Jushanwan, Jinting Town, Wuzhong District |
| 8 | Business scope: macaque, *Macaca fascicularis*, beagle |
| 9 | Under the "Animal Epidemic Prevention" provides, upon examination, qualified animal epidemic prevention |
| 10 | Issuing authority (stamp) |
| 11 | Date: September 6, 2010 |
| 12 | Submit report on the conditions of animal epidemic prevention and quarantine system implementation annually at the end of August |
| 13 | Supervision and inspection |
| 14 | 2010 qualified auditing date: August 26, 2011 |
| 15 | 2010 qualified auditing authority: Supervisor, The Ministry of Agriculture of the People's Republic of China |

### 2.3.3 Approval Permit for the Management of Wildlife and Products in Jiangsu Province


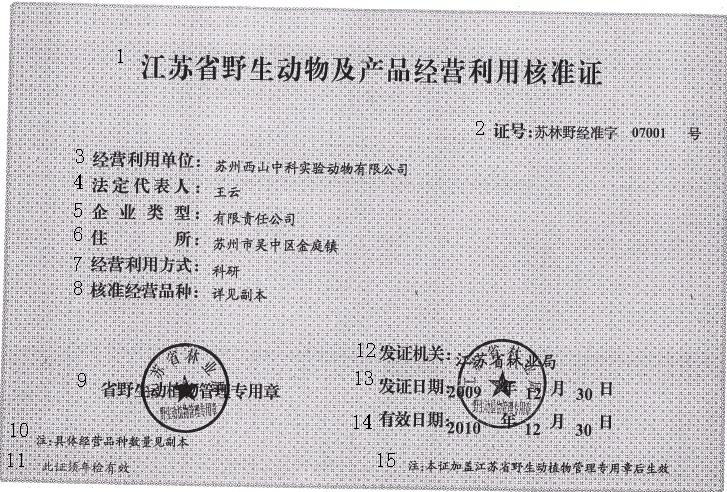


| **Item** | **English translation** |
| --- | --- |
| 1 | Approval Permit for the Management of Wildlife and Products in Jiangsu Province |
| 2 | Forest Bureau Permit No: 07001 |
| 3 | Business units: Suzhou Xishan Zhongke Laboratory Animal Co., Ltd. |
| 4 | Legal representative: Yun Wang |
| 5 | Business Type: Limited liability company |
| 6 | Address: Jinting Town, Wuzhong District, Suzhou Province |
| 7 | Business use: scientific research |
| 8 | Approval of product variety: See the copy |
| 9 | Provincial Wildlife Management Seal |
| 10 | Note: the number of specific business types to see the copy |
| 11 | This certificate represents a valid inspection |
| 12 | Issuing authority: Forestry Bureau in Jiangsu Province |
| 13 | Date of issue: Dec 30, 2009 |
| 14 | Effective Date: Dec 30, 2010 |
| 15 | Note: This card is valid only with the Provincial Wildlife Management Seal |

### 2.3.4 Business License


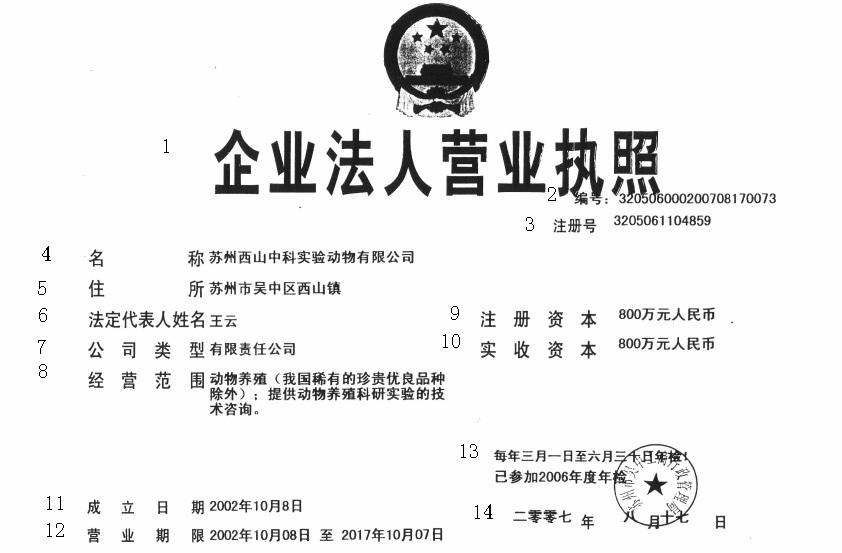


| **Item** | **English translation** |
| --- | --- |
| 1 | Business license |
| 2 | Business license No.: [see # above] |
| 3 | Registration number: [see # above] |
| 4 | Name: Suzhou Xishan Zhongke Laboratory Animal Co., Ltd. |
| 5 | Address：Jinting Town, Wuzhong District, Suzhou Province |
| 6 | Legal representative：Yun Wang |
| 7 | Business Type: Limited liability company |
| 8 | Business Scope: Animal breeding (except China's rare breeds); providing technical advice on experimental animal breeding technology |
| 9 | Registered capital: 8 million RMB |
| 10 | Paid-in capital: 8 million RMB |
| 11 | Establishment date ：Oct 8, 2002 |
| 12 | Business period: From Oct 8, 2002 to Oct 7, 2017 |
| 13 | Annual inspection (conducted from March 1 to June 30) |
| 14 | Aug 17, 2007 |

## Inventory

### [
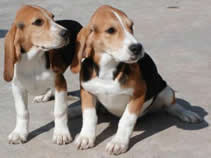
](http://www.szxszk.com/animal.do?id=3)2.4.1 Beagle

The beagle is a breed of small hound developed in Great Britain during the early 19^th^ century. Among dog breeds, beagles are internationally recognized in laboratory research. A typical adult beagle weighs 7-10 kg and spans 30-40 cm in length. Beagles are gentle in disposition, and easy to tame. They reach sexual maturity at 7-12 months and produce large litters. The Company holds 2000 high-quality beagles bred in closed colonies for purposes of scientific research.

### [2.4.2 Macaca fascicularis](http://www.szxszk.com/animal.do?id=2)


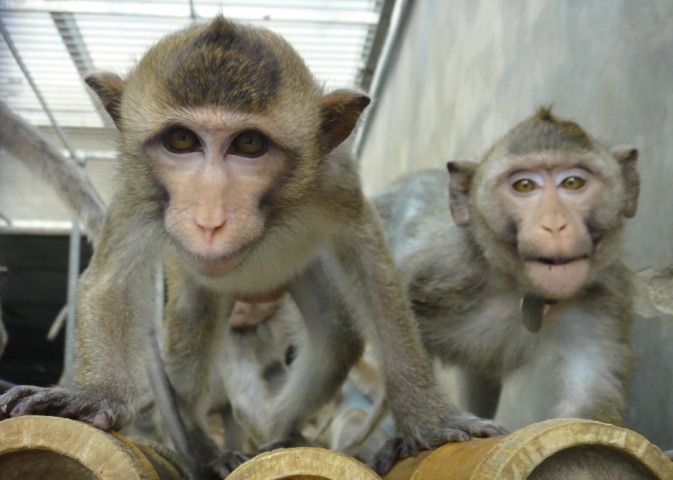
Cynomolgus monkeys (*Macaca fascicularis,* also known as the long-tailed or crab-eating macaque) are native to Thailand, Laos, Vietnam, Cambodia, Myanmar, Malaysia, Indonesia, the Philippines, the Andaman and Nicobar islands, and many small islands in Southeast Asia. They inhabit tropical rain forests, mangrove swamps, banks of tidal rivers and other tropical islands and shores. They commonly forage for crabs and shellfish on seashores after the tide has ebbed.

Cynomolgus monkeys are smaller than closely-related rhesus monkeys. The tail length is equal to or greater than the body length. Adult male and female cynomolgus monkeys weigh about 5 kg and 3 kg, respectively. More than 7000 high-quality cynomolgus monkeys (with clearly documented genetic backgrounds) are currently housed at the Company.

### [
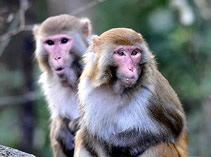
](http://www.szxszk.com/animal.do?id=1)2.4.3 Macaque

Macaque is the generic term for all 22 species of macaque monkeys (Genus *Macaca*). Aside from humans, macaque monkeys are the most widespread primate genus, ranging from Japan across Asia to North Africa. Of the 22 macaque species, the rhesus monkey (*Macaca mulatta*) is the most widely distributed, most numerous and most widely employed in laboratory studies. Rhesus monkeys are indigenous to Afghanistan, Pakistan, north India, Nepal, Bangladesh, Burma, Thailand, Vietnam, China's Guangxi and surrounding areas. The length of the tail is about half that of the body. Adult male and female rhesus monkeys weigh 5-10 kg and 4-7 kg, respectively. More than 1000 high-quality rhesus monkeys (with clearly documented genetic backgrounds) are currently housed at the Company.

# 3. Animal Welfare

Animal welfare can be segregated into five (5) basic elements:

- Physiological: attending to the subject's hunger and thirst
- Environmental: attending to the subject's housing
- Health & safety: attending to the subject's illnesses and injuries
- Behavioral: ensuring the subject's freedom within the environment
- Psychological: alleviating the subject's fear and anxiety of mind

## 3.1 Staff Assignment

### 3.1.1 Administrative Personnel

The Company has administrative personnel on staff who oversees the facilities. The administrators are required to be familiar with the physiological and zoological properties of the experimental animals, as well as some basic relevant veterinary knowledge. They are also required to manage and regulate daily breeding and quarantine activities, In addition, the administrators are required to be conscientious and earnest in their job duties.

### 3.1.2 Veterinarians

Full-time veterinarians are employed in the feeding base of the Company. The chief veterinarian is required to be an experienced professional veterinary doctor. The primary tasks of the veterinary staff are to prevent and treat disease, as well as assist the administrative personnel in their duties．

### 3.1.3 Feeders

The feeders are required to be healthy and to have completed a middle school education. According to the Regulation on Management of Laboratory Animals, one who is engaged in breeding laboratory primates should have received specialty training and passed certain examinations before starting work. The feeders also receive physical examinations before starting work and undergo repeat examinations at least once a year thereafter. Those carrying tuberculosis and/or pathogenic bacteria are forbidden within the breeding areas. Feeders suffering from epidemic diseases are not permitted to work.

## 3.2 Breeding Management

- The feeding base is located in an area with excellent air quality and quiet natural conditions, far from residential areas and main roads.
- Animal housing is well-ventilated, well-lit, clean, and dry. The internal wall is smooth with arched corners to enable thorough washing and sterilization. The internal wall is made from erosion-resistant, non-reflecting and impact-resistant materials, which are not susceptible to peeling. The ceiling is water-resistant and erosion-resistant. The roof was equipped with ventilation and lighting equipment. Heating equipment is installed for warming the environment during the fall and winter seasons．
- The houses for the laboratory group are maintained within the temperature range of 18-29℃ with a relative humidity ranging from 40-70% (varying with natural seasonality). In the houses for artificial breeding, the temperature range is maintained at 20℃-25℃ with a humidity ranging from 40-60%. There is ventilating and heating equipment installed to continually maintain and warm the air circulation．
- The breeding area is closely managed with fences enclosing the area. The area is divided into four (4) subareas: reproducing, growing, isolating and quarantine. In addition, there are several auxiliary rooms, such as feeding units, feed storage room, veterinarian room and treatment room．
- For the environmental indexes for breeding of primate laboratory animals, see below:

| **Item** | **Index** | |
| --- | --- | --- |
|  | Open System | Barrier System |
| Temperature (℃) | 16-29 | 18-29 |
| Relative humidity (％) | 40-70 | 40-70 |
| Clean degree of air (grade) |  | 10000 |
| Number of bacteria post-cleaning (bacteria/plate-hour, £) | 30 | 3 |
| Concentration of ammonia (mg/m3, £) | 14 | 14 |
| Working illumination (Lx) | 150-300 | 150-300 |
| Day-night cycle (h) | 12／12 or 10／14 | 12／12 or 10／14 |

Note: The ammonia concentration indicated in this form refers to an ambulatory index

## 3.3 Accommodation and Environment

### 3.3.1 Free enclosures

The design and equipment in the free enclosure was established on the basis of the animals’ comfort. This principle is embodied in providing comfortable conditions and sufficient space so the animals can live a free and active life. Units possessing surplus funds also equip bars, swings and other amenities in the free enclosures. In essence, free enclosures of primate laboratory animals are suitable for the subjects’ living, growing, and reproducing, as well as protective of their safety and health. On the basis of different breeding styles, two types of free enclosures were established: cage-breeding houses and room-breeding houses．

### 3.3.2 Cage-breeding houses

- Cage-breeding houses are spacious and bright, with sliding glass ventilating windows on the ceiling that allow external air and sunlight．Windows on both sides of the houses are installed for ventilation, and externally-installed barbed wiring acts as a barrier against the animals’ escape．As the breeding houses are subject to frequent washing, the floor is made from water-resistant, friction-resistant, and erosion-resistant materials．There is a 15 cm-high capstone at the interface between the floor and the wall; corners are arched．The floor is slightly inclined with a curved drainage outlet．
- Each cage-breeding house contains several cages, each measuring no smaller than 60 cm×60 cm×80 cm．Sealed with smooth, acid-and-alkali-resistant, erosion-resistant, and easy-to-sterilize steel rods, cages have a smooth interior surface, free from burrs and cracks．Each cage has a feed box and a water-canister installed．The cages were assorted linearly, with clapboards inserted between cages to prevent animals from assaulting one another．The clapboards can be pulled back-and-forth, so that the animal can be arrested internally．Spring-fastened zipper is installed on the front face of the door to allow better observation the animals．The base of the cage is placed 60 cm above the ground in order to prevent animals from retrieving contaminated foods from the ground．In addition, a sloped waste flume with a smooth surface is set under the cage which is convenient for cleaning; feces and urine can be easily washed to the main drainage outlet．The flume is kept clean, and was scrubbed down when necessary．
- Cage-breeding houses are generally employed for animals during quarantine or experiments.

### 3.3.3 Room-breeding houses

- Room-breeding houses are divided into internal and external chambers. The internal chamber is equipped with ventilating and heating equipment. There are also shelves installed so the animals can rest. The external chamber is a closed iron-fence structure exposed to the external environment, which has shelves and swings installed to support the animals’ outdoor activities．
- The area ratio of internal to external chamber ranges from 1:1.5-1:2.5. The combined area of one internal and one external chamber is 20-25 square meters, with a small door connecting the two chambers. The external chamber has an accessory pathway serving as an entry/exit point for personnel. The flooring in both chambers is slide-proof, wear-resistant, free-from-cracks and non-penetrable. The slope of the floor is slightly inclined for drainage purposes. Both internal and external chamber walls are smooth and neat, and the doors are water-resistant, erosion-proof, reflection-free, and impact-resistant. The corners are arched for the sake of washing and cleaning．
- Two groups of monkeys are raised in these houses; one is the reproductive monkey group and the other is the growing monkey group. There are, on average, eight (8) reproductive group monkeys per house (to approximate a female:male ratio of 7:1); in contrast, there are 8-13 individuals per house in the growing monkey group．

## 3.4 Animal Feeds and Water

- The feeds are kept clean and free from impurities, strange tastes, molds, fermentation, insects, and mice. Additives, such as antibiotics, antiseptics, insecticides, pigments, growth-promoting agents, and hormones cannot be added without official permission．
- The feeds are formulated considering the animals’ physiological properties, dietary habits, and oral adaptability. For example, monkeys consume a primarily vegetarian diet, so they favor fruits, vegetables, and corns. Therefore, it is important to make a rational feed formula based on these dietary habits. In order to meet the nutritional requirements of monkeys, unprocessed proteins account for at least 16% of adult monkey feed. This percentage is even higher (18-20%) for juvenile monkeys. Attention is also paid to the ratio of calcium to phosphorus and Vitamin C supplementation. In order to meet these strict dietary standards, the formula is designed by nutritional experts so the daily diet can be formulated in response to different feed types, seasonal alternations, and alterations in feed resources. Nutrient contents are also checked and measured periodically．
- Drinking water for the animals reaches or exceeds the standards of urban drinking water．Feeders are alert to notice any quality change in the water．There equipped with auto-drinkers inside the cages．

## 3.5 Tools

- Several tools are used in breeding animals, including pots, bowls, spoons, basins, barrels, and brooms．These tools are cleaned and sterilized in a timely manner, utilized on a cage-specific basis, and segregated by tool category．

## 3.6 Veterinary Care

Complete animal husbandry and veterinary care was provided daily.

## 3.7 Euthanasia of Non-human Primates

Animals are typically euthanized at the end of a study for the purpose of sample collection or post-mortem examination. Chongqing Medical University euthanasia guidelines, summarized in the [table of methods](http://research.uiowa.edu/animal/?get=euthanasia#Table%20of%20Euthanasia%20Methods), closely resemble those previously established by the American Veterinary Medical Association Panel on Euthanasia. Euthanasia techniques must be reviewed and approved by the Institutional Animal Care and Use Committee (IACUC). The appropriateness of the techniques employed may vary from species to species.

Euthanasia is carried out by personnel properly trained in the procedure being used. Measures are taken to ensure that euthanasia is performed in a way that minimizes reactions among other animals that may be present. Distress vocalizations, fearful behavior, and release of certain odors or pheromones by a frightened animal may cause anxiety and apprehension in other animals. Therefore, when possible, animals are not exposed to euthanasia of other subjects, especially of their own species. The resultant distress may lead to physiologic changes in other animals, which may affect later research results.

Careful handling of animals is of the utmost importance during the procedure in order to minimize distress for both the animal and handler. Appropriately conducted procedures which render the cerebral cortex nonfunctional by means of hypoxia or drug-induced anesthesia eliminate the animal's perception of pain. Proper euthanasia includes a follow-up exam to confirm the absence of a heartbeat to confirm death.

The following table summarizes the methods and procedures which are accepted by the IACUC for humane killing of animals. In general, these are the same as recommendations of the American Veterinary Medical Association (AVMA) Panel on Euthanasia, 2000. Also, a list of unacceptable methods deemed inhumane or dangerous to personnel and to other animals is included. The recommended barbiturate overdose for primates is used as noted below.

| **Method of euthanasia** | **Nonhuman primates** |
| --- | --- |
| Carbon dioxide | Unacceptable method of euthanasia |
| Barbiturate overdose (iv) | Method of choice |
| Barbiturate overdose (ip) | Unacceptable method of euthanasia |
| Anesthetic overdose | Method of choice |
| Exsanguination in anesthetized animal | Other acceptable method |
| KCL (iv) in anesthetized animal | Other acceptable method |
| Decapitation in sedated or anesthetized animal | Unacceptable method of euthanasia |
| Cervical dislocation in anesthetized or sedated animal | Unacceptable method of euthanasia |
| Decapitation in awake animal | Unacceptable method of euthanasia |
| Cervical dislocation in awake animal | Unacceptable method of euthanasia |
| Stunning in an awake animal | Unacceptable method of euthanasia |

Adapted from the report of the American Veterinary Medical Association Panel on Euthanasia (J. Am. Vet. Med. Assn. 218:669-695, 2001). The JAVMA article provides the rationale for these recommendations. The Chongqing Medical University Institutional Animal Care and Use Committee reviewed approved this table. These methods are in accordance with humane euthanasia as defined by the Federal Animal Welfare Act (54 FR 36112-36163). Methods always unacceptable in an awake animal include: potassium chloride, magnesium sulfate, strychnine, neuromuscular blocking agents, exsanguination, air embolism, and chloroform (due to its hazard to personnel).

#### Barbiturate euthanizing doses (mg/kg):

| **Species** | **IV route (mg/kg)** | **IP route (mg/kg)** |
| --- | --- | --- |
| Primate | 80 |  |

NOTE: Euthanasia with barbiturates usually requires approximately 3X the anesthetic dosage.

## 3.8 Sanitation and Epidemic Prevention

### 3.8.1 Sanitation and Epidemic Prevention

Sanitation and epidemic prevention are common issues in the breeding of experimental animals．Therefore, particular attention was given to these issues.

### 3.8.2 Sanitation

- The peripheral circumference around each free enclosure is cleaned weekly, and the waste is disposed of immediately．Areas vulnerable to mosquito and fly breeding are cleaned thoroughly.
- Houses are kept clean. The floor, corners, shelves, cages, food basins, and water boxes in the houses are cleaned daily; stool and residual foodstuff is disposed of and processed in the cesspit. In addition, the floor is sprayed and washed with 5% Lysol solution periodically.
- The animal field is also cleaned intermittently, so that the field is free from dirty water, useless grass, decaying foodstuffs, lichen, cobwebs and miscellaneous objects．
- Sanitary wares and breeding tools are used on a cage-specific basis and are not cross utilized. These tools are cleaned and sterilized immediately after use．
- Tools and instruments in the preparing room are cleaned and sterilized periodically．The staff actively kills rats, flies, mosquitoes and other pests in order to prevent outbreaks of arthropod-borne infectious diseases．
- Before entering the animal's area, both feeders and administrators dawn work clothes, Russian boots, masks and working hats．Boots and hats are assigned on an individual basis and sterilized in a timely manner. After work, the feeders and administrators clean and sterilize their hands thoroughly. If traumatic accidents happen to occur, the wounded person is treated immediately to prevent zoonoses．

### 3.8.3 Epidemic Prevention

- Objects in the monkey houses cannot be taken out of the houses before sterilization and vice-versa．Persons who are not members of the working staff are not permitted to enter the animal area without permission．If these persons must enter, they must change their clothes and sterilize themselves first, and then enter with certain personnel．
- Generally, sterilization was carried out once every 3 days．In summer and rainy seasons, sterilization was conducted every other day．Under special conditions (such as the prevalence of some infectious agent), sterilization can be done once daily or according to the request of veterinary staff.
- In front of the breeding farm, there is both a sterilizing room and a sterilizing pool．The sterilizing room is equipped with U.V. sterilizing lamps. In the pool, there is a sterilizing fluid (5% Lysol solution, which is in an effective concentration)．Besides, it was changed once a week．People who enter the breeding room must take sterilization before．
- Prophylactic sterilization was carried out in a timely manner. Tools and containers that are regularly used in the animal houses, including feed barrels, feed basins, food boxes, and water boxes, were soaked in 0.1-0.5% new antamon solution at least once a week．Monkey houses, monkey cages, trapping nets, and the instrument rooms were sprayed with 2% peroxyacetic acid as frequently as once every two weeks．After use, cleaning implements like brooms, mops, and drying cloths were soaked in 0．1% new antamon solution and then washed with water and air-dried before their next use．
- The following cages are washed and sterilized strictly, and checked by correlative microbe examinations before re-use: cages of sick monkeys, cages of dead monkeys, cages or houses that have been left vacant for an extended period of time．
- The breeding farm has isolated therapeutic room to quarantine and treat animals with infectious diseases to prevent such diseases from spreading．
- If there appears to be infected animals or animals die from unknown causes, all objects involved and surrounding areas are sterilized．After treating these animals, these cages are incinerated and the houses are sterilized thoroughly．These houses are left vacant for at least three months post-sterilization．
- Animals that die from diseases were delivered to veterinary staff for autopsy．Post-autopsy, the bodies are incinerated or buried．The autopsy site and the site of death are sterilized immediately．
- Newly imported animals are not allowed to enter the houses before quarantine．During quarantine, the animals are kept in special houses in a separate area．The quarantine period lasts for at least three (3) months. Only animals that are confirmed to be disease-free can be bred together．
- Green feeds are cleaned first, soaked in 0.01% potassium hypermanganate solution for more than 5 minutes, and then washed before feeding．
- The cages and tools used to transport animals are strictly sterilized before either entering or leaving the animal field．
- Rats, flies and other pests are eliminated periodically in order to prevent transmission of disease．

## 3.9 Feeding

### 3.9.1 Feeding method

Feeding is conducted in a timely and orderly manner, about two or three times per day．Each monkey is given about 300-400 grams of food per day．Observations on picking food is carried out during breeding time under the premise of not interrupting the subjects' picking activity．

### 3.9.2 Sanitation of feeds

Granular feeds that are processed in the workshop are stored away from possible contamination．All green feeds are kept fresh and mold-free; vegetables and fruits are cleaned, sterilized, and air-dried before feeding, thereby preventing intestinal infections or pesticide poisoning．Drinking boxes are cleaned and sterilized daily to ensure the cleanliness of the animals' drinking water．

## Recording of Observations

Recording of observations of monkeys are made according to the following:

- Feeding status: Do the animals display a normal appetite? Are the animals fastidious in feeding? Do they reject certain foods, or do they prefer some foods over others? Are there some monkeys which fail to feed adequately due to physical size or weakness?
- Mental status: Are the animals alert and active? Do they lower their heads and hug their bellies, or lie on the ground? Are there visible signs of altered affect, twitching, poor coordination and/or quadriplegia?
- GI and renal status：What is the color, shape, odor, frequency, water content, and amount of feces? Is there pus and/or blood in the stool? Does the stool emit a foul-smelling odor? What is the color, odor, frequency, and amount of urine? Is there vomiting, excessive thirst, abdominal distension, or diarrhea?
- Skin and joints：Are there signs of biting, trauma, dishevelment, or depilation? Is there any swelling or redness in the extremities and/or joints?
- Bleeding：What is the amount and color of blood on the floor? Is it from trauma, bloody stools, menstruation, miscarriage, or postnatal bleeding?
- Mating, pregnancy, and delivery: Is the female to male ratio reasonable? Is there any monkey deceived or abandoned by the group? What is the timing of the last menstrual cycle, copulation, pregnancy and delivery?
- Quarantine：Were animals quarantined for injuries or illnesses? What was the diagnosis, prognosis and treatment course/duration? What categories of drugs were administered? What was the outcome?
- Environmental controls: What is the temperature, humidity, ventilation status, ammonia concentration, and population density of the houses?

## 3.11 Management of Special Monkey Groups

### 3.11.1 Management of newly imported monkeys

- Initial check-up: Exogenous monkeys are first examined and weighed on arrival．Age, sex, weight, and results of clinical examinations are recorded in detail．
- Quarantine: See section 3．1 for the methods and procedure of quarantine.
- Adaptive breeding：In order to aid the newly imported monkeys in accommodating themselves to new conditions, adaptive breeding is required．Generally, such breeding should last for no less than three (3) months．This procedure is administered by certain specially-trained feeders or lab personnel. The content of the feeds is gradually adjusted to match the breeding requirements of the monkey groups．
- Climate and air management：The temperature of houses is kept at approximately 15℃ in winter and spring. Sudden changes in temperature were avoided to prevent pneumonia．Ventilation was carried out to prevent respiratory infections．
- Prophylactic treatment：Attention is paid to signs of common infectious diseases, including pneumonia and dysentery．Steps to improve prevention, treatment, and sterilization are taken. Drugs are given prophylactically．
- After quarantine and adaptive breeding, monkeys are bred in categories：
  - Monkeys that are going to be sent out: bred in small groups or in a single cage．
  - Pregnant monkeys ：sent to special cages and given better nutrition．
  - Stud monkeys ：bred in large cages (select healthy male monkeys from 3.5 to 12 years)．
  - Infant monkeys：raised by their mother or sent to infant rooms to be fed with artificial milk within six (6) months of age．
  - Sick monkeys：sent to the treatment room immediately for quarantine and treatment.

### 3.11.2 Management of stud monkeys

- A breeding pattern, termed imperial-harem-like reproduction, is commonly adopted. Monkey groups that are bred together are examined frequently．If there appears to be monkeys that are afraid of picking foodstuff, than these monkeys are picked out immediately as all monkeys should maintain normal relationships among the groups. Alterations in inter-group relationships without comprehensive thought are avoided as to prevent trauma, abortion, and premature delivery caused by conflict．
- Proteins account for 20% of the daily diet, accompanied by proper amounts of Vitamin C and other elements, as well as abundant green feeds like fruits and vegetables．
- Unnecessary disturbances are minimized as much as possible under non-special conditions．
- Unmatchable relationships are ended promptly by detecting and treating biting injury as early as possible. For monkeys with portent abortion, prompt treatment is also done （pregnendione 10-15 mg, VE 10 mg, Qd for 3 successive days).
- Sick monkeys are observed for abnormal manifestations．If any, appropriate treatment is given in a timely manner, and a veterinary report is filed．Besides, a tuberculin test is taken at least once a year．

### 3.11.3 Management of juvenile monkeys post-lactation

- Juvenile monkeys cease lactation at the age of 4-6 months．At that time, they are sent to the grown-up room to begin artificial feeding, and are assigned serial numbers and name plates. These identifiers are recorded in official files．
- Post-lactation juvenile monkeys are usually raised in cages, generally two monkeys per cage．The temperature of the grown-up room was maintained at no less than 15℃．
- Considering the nutritional requirements of juvenile monkeys, a feeding pattern of more frequent meals and smaller amounts is highly evaluated．In addition, a complement of 10 g milk powders, and a proper amount of cod-liver oil, yeast powder, and VC reagent, are added．
- Common conditions affecting juvenile monkeys include pneumonia, diarrhea and vomiting．In order to reduce the incidence of these conditions, the manager controls the temperature, food amount, and environment around the animals．

### 3.11.4 Management for sick monkeys

- First, sick monkeys are picked out as soon as possible, and placed into a treatment room for necessary quarantine and therapy．Attention is paid to fluid and electrolyte balance．
- Feeding of sick monkeys is in accord with both nutritional and therapeutic requirements through regulating the nature and amount of feeds．
- Persons assisting in feeding sick monkeys have mastered the relevant veterinary knowledge and corresponding therapeutic techniques to aid the veterinarian implement treatment.
- The treatment room is kept neat and clean at a relatively stable temperature．There is a sterilizing plate at the door. The floor was sterilized twice per week, in addition to an overall room sterilization weekly．All instruments and articles are sterilized immediately after use, then wiped, air-dried and replaced in an orderly manner．

## 3.12 Quality Control and Health Monitoring

## 3.12.1 Assessment of Health Conditions

Health conditions of the animals have a direct impact on experimental results. The general appearance and behavior of the monkeys is regularly assessed by the feeders including: a full body developed normally, a thick shiny coat, eyes bright and responsive, and a good appetite. In addition, feeders carefully check each monkey's body, including the following items:

- Eyes: pupil clarity, ocular discharge, eyelid inflammation.
- Ears: ear secretions, structural defects.
- Nose: serous, mucinous, and purulent exudates
- Skin: trauma, abscesses, scale and eczema
- Head and neck: correct posture (if skewed, often indicates inner ear inflammation)
- Legs: musculoskeletal development; fractures, deformations and osteoarthritis
- Gastrointestinal: vomiting, diarrhea, constipation, whether the perianal region is clean
- Nervous: tremor, paralysis and other symptoms
- Cardiopulmonary: auscultation to check and record breathing and heartbeat with special occasions for X-ray testing

### 3.12.2 Genetic quality standards

- The experimental monkey is an animal with a clear genetic heritage, so breeding populations can be appropriately established.
- In order to establish a complete breeding pedigree, monkeys are carefully numbered and listed to produce a breeding record of individuals and groups

### 3.12.3 Microbiological quality standards

- Normal animals: Primates must meet microbiology control requirements in order to prevent introducing major zoonotic pathogens that can cause serious harm to the health of animal populations. Therefore, seven (7) pathogens are tested including: Herpes B virus (HBV), Salmonella (*Salmonella*), shigellosis (*Shigella*), Mycobacterium tuberculosis (*Mycobacterium tuberculosis*), skin fungus (dermal fungi), parasites (Ectoparasites), and toxoplasmosis (*Toxoplasma gondii*).
- Specific pathogen free animals (SPF animals): Aside from normal animals, SPF animals are free of the seven (7) aforementioned pathogens, but are also free of other infectious pathogens. SPF primates, in addition to the aforementioned checks, are tested for three (3) kinds of serum antibodies: Simian Retrovirus D (SRV), Simian Immunodeficiency Virus (SIV), and Simian T-lymphotropic Virus Type l (STLV-1). The monkeys who tested negative for these antibodies are then enrolled into SPF group. In addition, the bacteria *Yesinia enterocolitica* and *Campylobacter jejuni* and the parasites Helminth spp., Entamoeba spp., Plasmodium spp., and Flagellate spp. are ruled out. Strict feeding and microbiological control to prevent foreign pathogenic infection and regular tests to exclude HIV-positive or suspect animals are also conducted. SPF animals meet the highest international standards for experimental laboratory animals.

### 3.12.4 Export quality standards

Before exportation off-site, health inspection including tests for TB, enteric pathogens (Shigella, Salmonella), parasites, and intestinal worms is conducted. If specially requested, there can be further examinations, including tests for BV, SRV, SIV, STLV, etc.

### 3.12.5 Monitoring procedures and requirements

- Monitoring procedures
  - Primate laboratory animals (cage number, monkey no.)
  - Tuberculin antibody test
  - Blood virus antibody test
  - Blood parasite examination
  - Biochemical blood tests
  - Hair and skin tests for fungi
  - Anorectal examination for intestinal pathogens
  - Stool parasite examination
- Standard monitoring requirements for primate laboratory animals
  - Feeding monkeys: different levels of monitoring as required by the standards set by the particular project
  - Normal animals: tested more than once a year
  - SPF animals: tested once every quarter with a comprehensive examination for virus detection; other tests were conducted twice a year
  - Exported monkeys: quarantined for 45 days with a comprehensive inspection. If users have special requirements, additional inspection items can be added (such as blood examinations)
  - Newly imported monkeys: quarantined for three (3) months

### 3.12.6 Micro-organism and parasite inspection

- For the examination procedures and methods applied to detect microbes and parasites, see the 2001 version of the Chinese National Standards (GB l4922.1-2001；GB l4922.2-2001)

# References

1. Noldus L (1991) The Observer: A software system for collection and analysis of observational data. Behavior Research Methods, Instruments & Computers 23: 415-429.

2. Noldus IT (2003) The Observer: Professional system for collection, analysis, presentation

and management of observational data. Reference Manual, Version 50 Wageningen, The Netherlands:.

3. Regan T (1983) The Case for Animal Rights. University of California Press,.

4. John McGlone SFea (Jan 2010) Guide for the care and use of Agricultural Animals in Research and Teaching.

5. Pich J, Carne X, Arnaiz JA, Gomez B, Trilla A, et al. (9362) Role of a research ethics committee in follow-up and publication of results. Lancet 361: 1015-1016.

6. Edwards SJL, Lilford RJ, Hewison J (7167) The ethics of randomised controlled trials from the perspectives of patients, the public, and healthcare professionals. BMJ 317: 1209-1212.

7. Vail A (2811) Experiences of a biostatistician on a U.K. Research Ethics Committee. Statistics in Medicine 17: 2811-2814.

8. Yank V, Rennie D (2835) Reporting of informed consent and ethics committee approval in clinical trials. Jama 287: 2835-2838.

9. Waldorf AKM, Rubens CE, Gravett MG (2011) Use of nonhuman primate models to investigate mechanisms of infection-associated preterm birth. BJOG: An International Journal of Obstetrics & Gynaecology 118: 136-144.

10. Olsson AIS, Sandoe P (2010) "What's wrong with my monkey?" Ethical perspectives on germline transgenesis in marmosets. Transgenic Research 19: 181-186.

11. Lin Cf LMSCCCYCM (2010) A comparison of problem-based learning and conventional teaching in nursing ethics education. Nursing ethics 17: 373-382.

12. McManamon RDVM (2008) Diagnostic Testing in Nonhuman Primates. Journal of Exotic Pet Medicine 17: 31-38.

13. Gross U (2008) Public opinion and the ethics of primate brain research. Nature 456: 443.

14. Tharyan P (2007) Ethics committees and clinical trials registration in India: opportunities, obligations, challenges and solutions. Indian Journal of Medical Ethics 4: 168-169.

15. Roux FA, Sai P, Deschamps J-Y (2007) Some ethical issues regarding xenotransfusion. Xenotransplantation 14: 217-221.

16. Quigley M (2007) Non-human primates: the appropriate subjects of biomedical research? Journal of Medical Ethics 33: 655-658.

17. Edwards SJ, Stone T, Swift T (2007) Differences between research ethics committees. International Journal of Technology Assessment in Health Care 23: 17-23.

18. Widdershoven GAM (2005) Euthanasia, Ethics and Public Policy. An Argument Against Legislation. [Book or Media Review].

19. Mathis R (2005) Ethics and evidence based medicine: fallibility and responsibility in clinical science. [Book or Media Review].

20. Kralik D, Warren J, Price K, Koch T, Pignone G (2005) The ethics of research using electronic mail discussion groups. Journal of Advanced Nursing 52: 537-545.

21. Jeffrey D (2005) The Ethics of Palliative Care: European Perspectives. [Book or Media Review].

22. Bailey J (2005) Non-human primates in medical research and drug development: a critical review. Biogenic Amines 19: 235-255.

23. Uzych L (2004) Public Health Law and Ethics: a Reader. [Book or Media Review].

24. Tong R (2004) Risk and Luck in Medical Ethics. [Book or Media Review].

25. Reed KE, Bidner LR (2004) Primate communities: Past, present, and possible future. American Journal of Physical Anthropology 39: 2-39.

26. Cook AL, St Claire M, Sams R (2004) Use of florfenicol in non-human primates. Journal of Medical Primatology 33: 127-133.

27. Lehman SM (2000) Primate Community Structure in Guyana: A Biogeographic Analysis. International Journal of Primatology 21: 333-351.

28. Paul C (1111) Health researchers' views of ethics committee functioning in New Zealand. New Zealand Medical Journal 113: 210-214.

29. Williamson T Ethics of assertive outreach (assertive community treatment teams). [Miscellaneous].

30. t Hart BAabc, Amor Sac The use of animal models to investigate the pathogenesis of neuroinflammatory disorders of the central nervous system. [Miscellaneous].

31. Strech DMDP, Hurst SMD, Danis MMD The Role of Ethics Committees and Ethics Consultation in Allocation Decisions: A 4-Stage Process. [Article].

32. Stirrat GM Ethics and evidence based surgery. [Report].

33. Steinke EEPRN Research Ethics, Informed Consent, and Participant Recruitment. [Article].

34. Spilker B Guide to Drug Development: A Comprehensive Review and Assessment.

35. Sorrell JMPRNF Listening in Thin Places: Ethics in the Care of Persons With Alzheimer's Disease. [Article].

36. Reiter-Theil S Ethics consultation on demand: concepts, practical experiences and a case study. [Article].

37. Reiser SJ, Heitman E Creating a course on ethics in the biological sciences. [Article].

38. Reiser SJ Overlooking ethics in the search for objectivity and misconduct in science. [Article].

39. Ramsey DJMDPMPH, Schmidt MLMD, Anderson-Shaw LDMAMSN Online Ethics Discussion Forum Facilitates Medical Center Clinical Ethics Case Reviews. [Article].

40. Rambur BDRN Ethics, Economics, and the Erosion of Physician Authority: A Leadership Role for Nurses. [Miscellaneous].

41. Prescott MJ, Jennings M Ethical and welfare implications of the acquisition and transport of non-human primates for use in research and testing. ATLA Alternatives to Laboratory Animals 32: 323-327.

42. Phaosavasdi S, Thaneepanichskul S, Tannirandorn Y, Thamkhantho M, Pruksapong C, et al. Animals and ethics. Journal of the Medical Association of Thailand 88: 287-293.

43. McCarthy J Principlism or narrative ethics: must we choose between them?. [Article].

44. Association AVM (2007) AVMA Guidelines on Euthanais.
